# *In Situ* Sustained Delivery of Tumor Cell‐Derived Extracellular Nanovesicles With Oncolytic Adenoviruses for Potentiating Cancer Immunotherapy

**DOI:** 10.1002/jev2.70222

**Published:** 2026-01-06

**Authors:** Tianye Wang, Sheng Zhao, Zao Ji, Zhonggui He, Zhenguo Cheng, Zhen Gu, Yuqi Zhang, Jin Sun, Funan Liu, Mengchi Sun

**Affiliations:** ^1^ Department of Surgical Oncology and General Surgery The First Hospital of China Medical University Shenyang China; ^2^ Department of General Surgery The First Hospital of Dalian Medical University Dalian China; ^3^ Key Laboratory of Precision Diagnosis and Treatment of Gastrointestinal tumors China Medical University Ministry of Education Shenyang China; ^4^ State Key Laboratory of Advanced Drug Delivery and Release Systems, School of Pharmacy Zhejiang University Hangzhou China; ^5^ Wuya College of Innovation Shenyang Pharmaceutical University Shenyang China; ^6^ Joint International Research Laboratory of Intelligent Drug Delivery Systems Ministry of Education Shenyang China; ^7^ Sino‐British Research Centre for Molecular Oncology, National Centre for International Research in Cell and Gene Therapy, School of Basic Medical Sciences Academy of Medical Sciences Zhengzhou University Zhengzhou China; ^8^ Liangzhu Laboratory Zhejiang University Medical Center Hangzhou China; ^9^ Jinhua Institute of Zhejiang University Jinhua China; ^10^ Department of General Surgery, Sir Run Run Shaw Hospital, School of Medicine Zhejiang University Hangzhou China; ^11^ MOE Key Laboratory of Macromolecular Synthesis and Functionalization, Department of Polymer Science and Engineering Zhejiang University Hangzhou China; ^12^ Department of Burns and Wound Care Center, Second Affiliated Hospital, School of Medicine Zhejiang University Hangzhou China; ^13^ Phase I Clinical Trails Center, The First Hospital China Medical University Shenyang China; ^14^ School of Pharmacy Shenyang Pharmaceutical University Shenyang China

**Keywords:** cancer immunotherapy, drug delivery, microneedle, oncolytic adenovirus, tumor cell‐derived extracellular nanovesicle

## Abstract

Oncolytic adenoviruses (OVs) can directly eliminate cancer cells and subsequently activate immune responses, exhibiting potent antitumor therapeutics. However, it was observed that the immune cells can also be lysed during viral treatment, evidently dampening the OVs‐mediated antitumor immune response. In this study, we develop a microneedle (MN)‐based *in situ* tumor cell‐derived extracellular nanovesicle (TDEV)‐cloaked OVs platform to enhance cancer immunotherapy and reduce immune cell exhaustion. In this platform, tumor cells pre‐infected with OVs are loaded into the upper reservoir of the MN device. Following the transdermal administration, the hollow MN would constantly facilitate the transport of *in situ* the generated TDEV‐encapsulating OVs into the tumor site for sustained delivery of OVs, which could subsequently infect cancer cells selectively rather than immune cells. Enhanced antigens triggered by improved intratumoral OVs killing can be presented by non‐exhausted dendritic cells, further evoking significant immunotherapeutic effects in both TC‐1‐hCD46 xenograft tumor‐bearing mice and postoperative tumor recurrence mice models.

## 1 Introduction

A desirable immunotherapy strategy currently in use for cancer is oncolytic virus‐based biotherapy, which selectively replicates and kills cancer cells (Shalhout et al. 2023; Ma et al. 2023; Zhao et al. 2024). Importantly, it can result in the massive release of tumor‐associated antigens following the lysis of tumor cells, subsequently recruiting immune cells such as dendritic cells (DCs) to infiltrate the tumor site (Sun et al. 2022; Ban et al. 2023; Huang et al. 2022). This allows for the capture, processing, and presentation of tumor antigens to T cells, leading to tumor‐specific T cell immunity (Wu et al. 2023; Xu et al. 2023). Oncolytic adenoviruses (OVs) are among the most commonly used oncolytic viruses due to their excellent safety and efficacy profiles (Lin et al. 2023; Lowenstein et al. 2025; Qian et al. 2025), whereas clinical outcomes with OVs have not met expectations (Todo et al. 2022; Toulmonde et al. 2022; Mathlouthi et al. 2024). In our previous studies, we observed that OVs not only lyse tumor cells but also deplete recruited DCs, which affected the efficacy of tumor‐specific T cell immunity.

Moreover, intratumoral injection of OVs (the predominant clinical delivery route) is hindered by high intratumoral pressure and physical barriers in solid tumors, limiting OVs distribution and therapeutic efficacy (Li et al. 2022; Chang et al. 2021). The development of oncolytic viral therapeutics capable of overcoming these limitations is, therefore, highly desirable for augmented cancer immunotherapy.

Tumor cell‐derived extracellular nanovesicles (TDEVs), ranging in size from approximately 40‐150 nm are nanoscale spherical lipid bilayer vesicles secreted by cells, which exhibit significant homologous cell uptake capabilities (Garcia‐Martin et al. 2022; Yao et al. 2025; Xu et al. 2024). It is well known that TDEVs expressed CD47 on their surface show homologously targeting ability and emit a signal of “Don't eat me” to evade detection and elimination by immune cells (Cheng et al. 2021; Xie et al. 2021; Cheng et al. 2023). Of note, maintaining the integrity and stability of TDEVs is crucial for exerting their functions (Huang et al. 2022; van Niel et al. 2022). In the available research, TDEVs have primarily been utilized as drug carriers through repetitive physical extrusion or electroporation for encapsulation (Huang et al. 2023; Carney et al. 2025; Luo et al. 2025). However, these cumbersome approaches may potentially dampen the protein integrity of TDEVs, affecting their biological functionality (Zhang et al. 2021; Wang et al. 2023).

**FIGURE 1:**
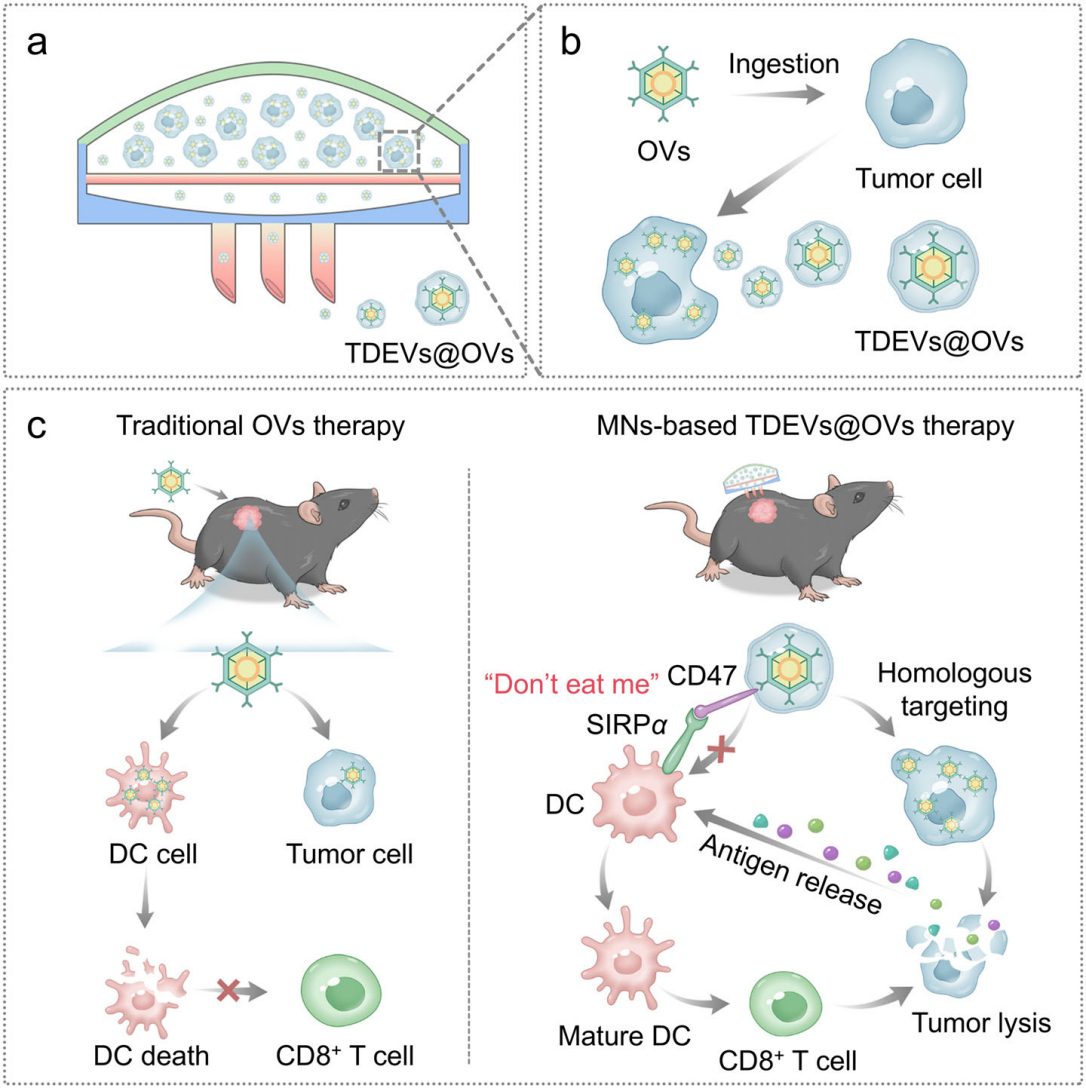
**MN‐based *in situ* TDEVs@OVs platform for boosting cancer immunotherapy and preventing immune cell exhaustion**. (a) Pre‐infected tumor cells loaded into the upper reservoir of hollow MNs and constant in situ releasing of TDEVs@OVs. (b) Schematic of OVs ingested by tumor cells and constant secretion of TDEVs@OVs. (c) MNs‐based in situ secretion of TDEVs@OVs for boosting cancer immunotherapy and preventing immune cells exhaustion.

To this end, we developed a microneedle (MN)‐based *in situ* TDEVs‐cloaked OVs (TDEVs@OVs) platform. In this technique, tumor cells pre‐infected with OVs were loaded into the upper reservoir of the MN device, and the intermediate polycarbonate membrane of the MN could prevent the passage of tumor cells while enabling a continuous release of TDEVs@OVs. Furthermore, the upper latex elastic membrane could provide a continuous contraction force to facilitate the diffusion of TDEVs@OVs through the hollow MN. The released TDEVs@OVs could home to homologous cancer cells while reducing undesirable uptake by DCs. The highly selective cytotoxicity against cancer cells as well as efficient prevention of DCs’ exhaustion would enhance DCs‐mediated T cell immunity and maximize the immunostimulatory potential of OVs. Moreover, the MN array can promote uniform drug distribution within the tumor to overcome the inherent physical barriers of the tumor, thus enhancing the antitumor efficacy of OVs (Figure 1a–c).

## 2 Results

### 2.1 Preparation and Characterization

**FIGURE 2:**
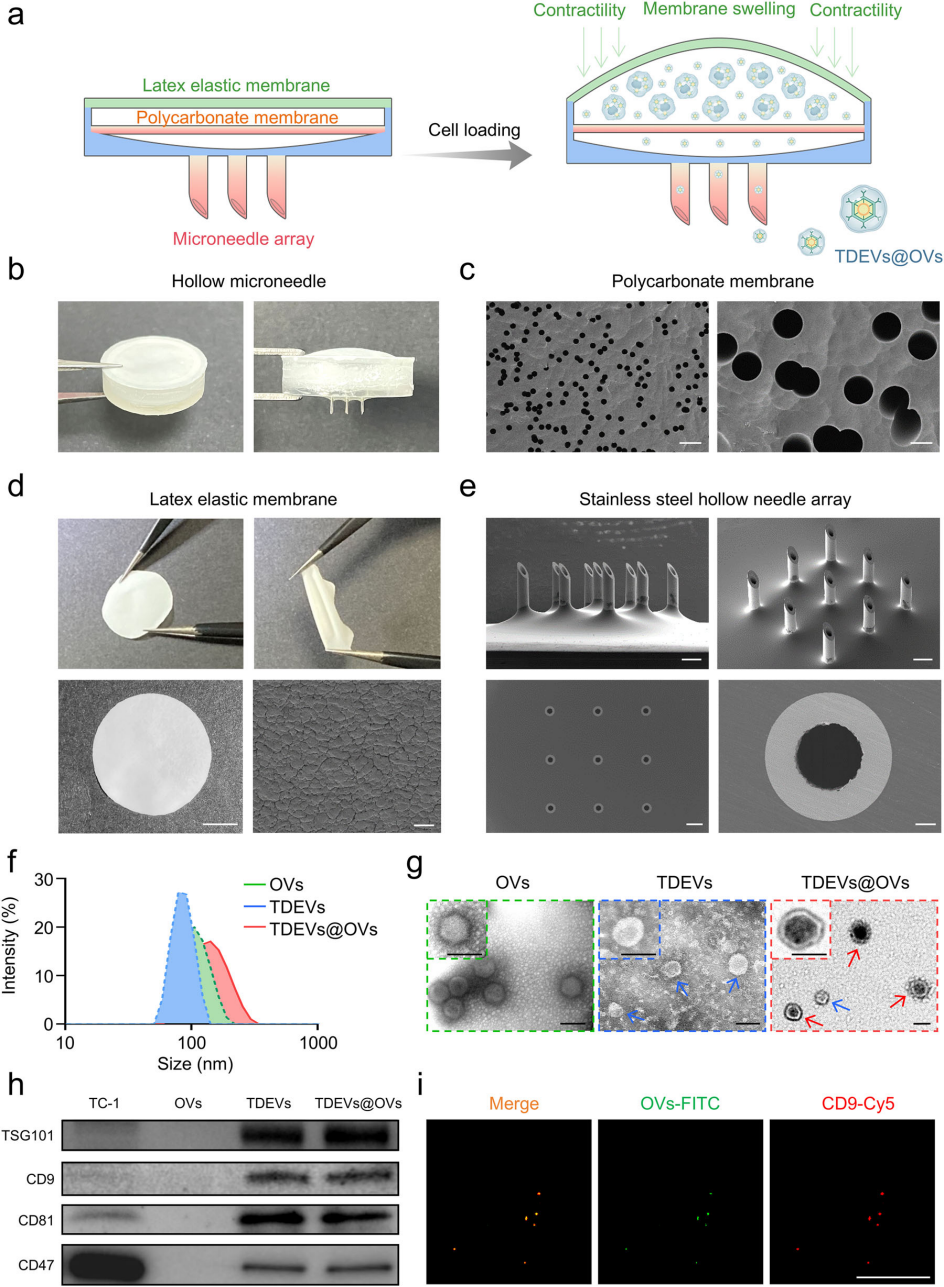
**Preparation and characterization of the MNs**. (a) Schematic of the MNs fabrication. (b) A representative picture of the MNs. (c) A representative picture of polycarbonate membrane; scale bars: 10 µm (left), 2 µm (right). (d) A representative picture of latex elastic membrane; scale bar: 5 mm (left), 20 µm (right). (e) Representative SEM images of the MN array (Stainless steel hollow needle); scale bar: 500 µm (upper and lower left), 50 µm (lower right). (f) Size distribution images of OVs, TDEVs, and TDEVs@OVs. (g) TEM of OVs, TDEVs, and TDEVs@OVs. Scale bar: 100 nm. Red arrow: TDEVs@OVs, blue arrow: TDEVs. (h) The expression of TDEVs‐related TSG101, CD9, CD81 proteins, and “Don't eat me” signal‐related CD47 proteins by western bolt analyses. (i) Colocalization of TDEVs@OVs by CLSM. OVs were labeled with FITC dye (green) while TDEVs was labeled with CD9‐Cy5 dye (red); scale bar: 100 µm.

In this study, we developed an MN‐based *in situ* TDEVs‐cloaked OVs platform. Mouse lung cancer cells (TC‐1) pre‐infected by OVs were loaded into the hollow MN, and TC‐1‐exocytosed TDEVs@OVs were released from the MN device for cancer immunotherapy. To fabricate the MN array, stainless steel hollow MN was first fixed to the polymethyl methacrylate (PMMA) base with holes machined by a numerical control machine (CNC). A representative MN patch was arranged in a 3 × 3 array, and each needle had approximately 1450 µm in height, 310 µm in base diameter, and 160 µm in inner diameter (Figure 2a–2b and Figure 2e). The microporous structure of the polycarbonate membrane of approximately 3 µm was validated using a scanning electron microscope (Figure 2c). The OVs were enclosed with a latex elastic membrane to enhance cell storage capabilities. Furthermore, the latex elastic membrane exhibited excellent deformation ability (Figure 2d), facilitating the transportation of the TDEVs@OVs solution.

Dynamic light scattering (DLS) and transmission electron microscopy (TEM) were utilized for the characterization of OVs, TDEVs, and TDEVs@OVs (Figure 2f–2g). The diameters of naked OVs and TDEVs were 98.15 ± 1.13 nm and 83.88 ± 5.08 nm, while TDEVs@OVs exhibited a diameter of 114.97 ± 2.45 nm. In contrast, TC‐1 cells were substantially larger by over 10 µm in diameter. This further supported the premise that TDEVs@OVs can successfully traverse polycarbonate membranes, whereas TC‐1 cells were effectively isolated in the upper reservoir of the hollow MN. Western blot results showed that, similar to the purified TDEVs, biomarkers TSG101, CD9, CD81, and a “Don't eat me” signal biomarker of CD47 in TDEVs were also detected in TDEVs@OVs (Figure 2h). To further prove that OVs were sheathed with TDEVs, a Cy5‐conjugated CD9 (a common biomarker for TDEVs) antibody was used to label TDEVs@OVs. As shown by immunofluorescent staining, colocalization of red CD9 fluorescence with green OVs fluorescence was observed in TDEVs@OVs (Figure 2i and Figure S1). Similarly, the colocalization of TDEVs with OVs was also observed by using another biomarker, CD81, which suggests the membrane sheathed on OVs was TDEVs (Figure S2). To further confirm the structural association between OVs and TDEVs, we performed an immunoprecipitation assay using an adenovirus‐specific anti‐hexon antibody. As shown in Figure S3, a high percentage of naked OVs were robustly precipitated, whereas the TDEVs@OVs group exhibited significantly lower precipitation efficiency, indicating that the viral capsid was shielded by the TDEVs. Notably, after disrupting the TDEV lipid bilayer with Triton X‐100, the precipitation efficiency of TDEVs@OVs by the anti‐hexon antibody was substantially increased, providing strong biochemical evidence for the encapsulation of OVs within TDEVs. Flow cytometry analysis using antibodies against CD9 and CD81 further demonstrated a strong association between OVs and TDEV markers (Figures S4–S6). The results indicated that a majority of OVs were positive for CD9 and CD81, providing additional evidence for the successful encapsulation of OVs within TDEVs.

Additionally, we assessed the OVs pre‐infected TC‐1 cell viability by CCK‐8 assay, which indicated that pre‐infected TC‐1 cells with a low viral dose of OVs did not exhibit significant cell death after 48 h (Figure S7). We loaded the OVs pre‐infected TC‐1 cells into the MN, and their live/dead viability was assessed by confocal laser scanning microscope (CLSM) after incubation at room temperature. The results demonstrated that OVs pre‐infected TC‐1 cells could survive for 48 h within the MN (Figure S8). Moreover, the OVs released from the MN were detected by polymerase chain reaction (PCR) (Figure S9). When the MN was loaded with naked OVs or OVs pre‐infected TC‐1 cells at a dose equivalent to 10^8^ pfu of OVs, the amount of OVs released from both sources was nearly identical at approximately 48 h.

### 2.2 *In Vitro* Evaluation of MN‐Based *In Situ* TDEVs‐Cloaked OVs

**FIGURE 3:**
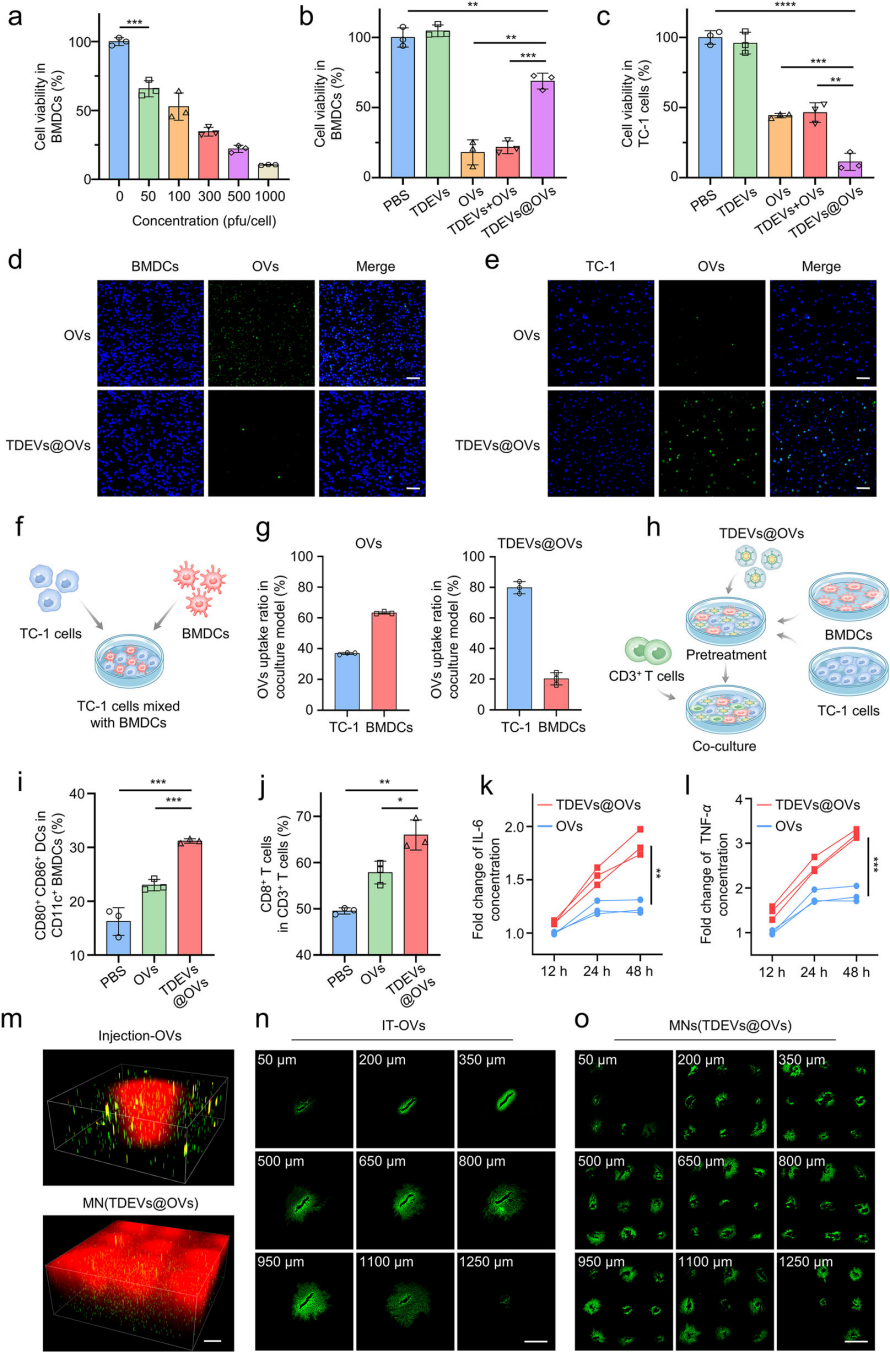
***In vitro* evaluation of MN‐based *in situ* TDEVs‐cloaked OVs**. (a) *In vitro* cytotoxicity of different concentration of OVs against BMDCs (*n* = 3). *In vitro* cytotoxicity of different samples against BMDCs (b) and TC‐1 cells (c) (*n* = 3). CLSM images of FITC‐labeled naked OVs and TDEVs@OVs endocytosed by BMDCs (d) and TC‐1 cells (**e**). Scale bar: 100 µm. (f, g) Flow cytometry evaluated the uptake rate of FITC‐labeled naked OVs and TDEVs@OVs in BMDCs‐TC‐1 cells co‐culture model. Schematic diagram of the co‐culture model (f) and results for flow cytometry analysis (*n* = 3). (h) Schematic diagram of the establishment of a TC‐1‐BMDCs‐CD3^+^ T cells co‐culture model. BMDCs were co‐cultured with TC‐1 cells and treated with naked OVs or TDEVs@OVs for 24 h. CD3^+^ T cells were added and cocultured for 48 h. The cells from the co‐culture model were collected for flow cytometry analysis. The results for flow cytometry analysis of DCs (CD11^+^CD80^+^CD86^+^) (i) and CD8^+^ T cells (CD3^+^CD8^+^) (j) in the TC‐1‐BMDCs‐CD3^+^ T cells co‐culture model (*n* = 3). (k, l) The cytokine secretion from BMDCs at 12, 24 and 48 h after treatment with naked OVs or TDEVs@OVs in BMDCs‐TC‐1 cells co‐culture model. Data are presented as fold change over the control group (*n* = 3). (m) CLSM images of OVs‐Cy5.5 (red) delivered by single‐needle injection or the MNs into a 3D TC‐1‐Dio (green) tumor model in vitro. Scale bar: 500 µm. Distribution of OVs‐FITC (green) within the TC‐1 tumor after single needle intratumoral injection (n) and the MNs insertion (**o**). Scale bar: 1 mm. Statistical significance was analyzed by unpaired *t* test. Data are presented as mean ± SD. *P*‐value: *P < 0.05, **P < 0.01, ***P < 0.001, ****P < 0.0001.

To assess the cytotoxicity of the OVs towards DCs, we first coincubated DC2.4 cell line with OVs at different concentrations. As displayed in Figure S10, OVs showed significant cytotoxicity against DCs. Consistently, we coincubated bone marrow‐derived DCs (BMDCs) collected from C57BL/6 mice with OVs at different concentrations, further exploring the cytotoxic capacity of the OVs against BMDCs. As shown in Figure 3a, OVs showed significant cytotoxicity towards BMDCs as well. However, in the bone marrow‐derived macrophage cells (BMDMs) and spleen‐derived CD3^+^ T cells, there was faint cytotoxicity observed at the same concentration of OVs (Figures S11–S12). Furthermore, we assessed the cytotoxic capacity against BMDCs and TC‐1 cells by co‐incubation with secreted TDEVs@OVs or naked OVs. As shown in Figure 3b, the group treated with TDEVs@OVs exhibited reduced cytotoxicity against BMDCs compared with the OVs group, indicating the protection of DCs from OVs‐induced cell death. On the contrary, the TDEVs@OVs‐treated group showed great cytotoxic capacity against TC‐1 cells in comparison to the OVs group, which suggested the selected cytotoxic capacity of TDEVs@OVs released from the MN (Figure 3c). Concurrently, we employed anti‐CD47 antibody blockade experiments to evaluate the cytotoxic effects of TDEVs@OVs versus naked OVs on both BMDCs and TC‐1 cells. As shown in Figure S13, blockade of CD47 significantly enhanced the cytotoxicity of TDEVs@OVs toward BMDCs. In contrast, the potent cytotoxic effect of TDEVs@OVs on TC‐1 cells was not appreciably altered and remained substantially higher than that of the naked OVs group. These results confirm that TDEVs@OVs can protect DCs from OVs‐induced cell death through the “Don't eat me” signaling mechanism.

We further investigated the cellular uptake mechanisms of TDEVs@OVs released from the MN. The results demonstrated that the uptake of TDEVs@OVs in BMDCs was significantly reduced compared to the naked OVs group. Conversely, the uptake of TDEVs@OVs in the TC‐1 cells was notably increased. In addition, we performed blockade experiments with anti‐CD47 antibody for further validation. Anti‐CD47 treatment markedly increased the uptake of TDEVs@OVs by BMDCs. In contrast, the uptake of TDEVs@OVs by TC‐1 cells was not appreciably affected and remained substantially higher than that of naked OVs (Figure 3d–3e and Figure S14). These findings are consistent with the cytotoxicity assay results. Moreover, to prove the selective uptake capability of the TDEVs@OVs in a multicellular system, we established a BMDCs‐TC‐1 cell co‐culture model *in vitro*, being employed to assess the uptake efficiency of OVs in BMDCs (CD45^+^CD11c^+^) and TC‐1 cells (CD45^−^CD11c^−^) through flow cytometry (Figure 3f and Figure S15). As presented in the result of flow cytometry analysis (Figure 3g), about 64% of the uptaken OVs were distributed in BMDCs and 36% in TC‐1 cells in the naked OVs‐treated group, while in the TDEVs@OVs‐treated group, about 20.2% of the OVs were taken up by BMDCs and 79.8% by TC‐1 cells. The results further confirmed that the TDEVs@OVs could enhance the uptake capacity by TC‐1 cells while escaping BMDCs.

Furthermore, the capacity of TDEVs@OVs to promote DCs maturation and immune activation was evaluated in a TC‐1‐BMDCs‐CD3^+^ T cells co‐culture model *in vitro*. BMDCs were co‐cultured with TC‐1 cells and treated with naked OVs or TDEVs@OVs collected from the MN. Following a 24 h incubation period, CD3^+^ T cells were introduced and cocultured for another 48 h (Figure 3h). Subsequently, cells from the co‐culture model were harvested for flow cytometry analysis. The results revealed that TDEVs@OVs exhibited greater efficacy in promoting the maturation of the DCs (CD11^+^CD80^+^CD86^+^) than naked OVs_._ Besides, the TDEVs@OVs‐treated group could activate CD8^+^ T cells, thereby inducing tumor‐specific T cell immunity (Figure 3i–3j and Figure S16). Moreover, it was observed that TDEVs@OVs could prevent DCs exhaustion, as evidenced by the prolonged and sustained secretion of proinflammatory cytokines (IL‐6 and TNF‐*α*) by BMDCs in the BMDCs‐TC‐1 cells co‐culture model (Figure 3k–3l).

In addition, to explore the uniform distribution of TDEVs@OVs by the MN array compared with single needle‐mediated injection of OVs, we developed an *in vitro* 3D Matrigel model containing tumor cells to mimic the solid tumor. The CLSM images showed that the MN promoted a wider distribution of OVs by the MN array structure, while the single needle‐based injection group was restricted to a confined space (Figure 3m). Then, we compared the intratumoral distribution of OVs through intratumoral injection and the MN‐mediated delivery in the TC‐1‐hCD46 xenograft tumor‐bearing mice. The CLSM image showed that OVs were confined within the applied region through intratumoral injection. In contrast, the MN‐treated group showed prominent tumor infiltration (Figure 3n–3o).

### 2.3 *In Vivo* Anti‐Tumor Efficacy and Immune Activation Capacity

**FIGURE 4:**
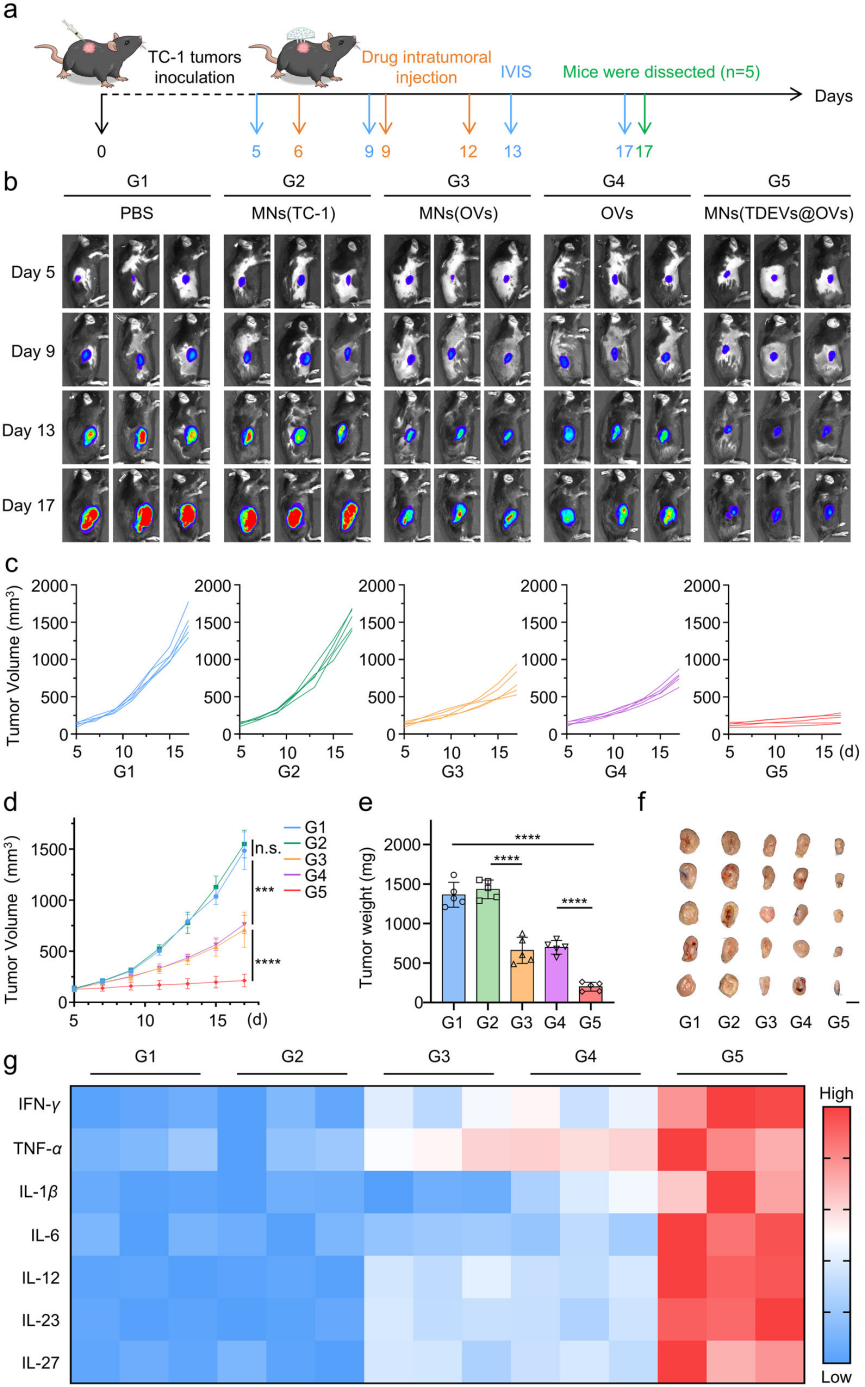
***In vivo* oncolytic efficacy of the MN‐based *in situ* TDEVs‐cloaked OVs**. (a) Schematic illustration of the antitumor activity of the MNs(TDEVs@OVs) using TC‐1‐hCD46 xenograft tumor‐bearing C57BL/6 female mice model. (b) Bioluminescence images of mice receiving treatments with different agents. (c) Individual tumor growth kinetics in different groups (*n* = 5). (d) Growth curve of tumor volume for TC‐1‐bearing mice after receiving treatments with different agents (*n* = 5). (e) Statistical graph of tumor weight of different treated groups (*n* = 5). (f) Images of representative tumors of different treated groups on the 17th day (*n* = 5). Scar bar: 1 cm. (g) The cytokines in the tumor were detected after different treatments, using the Luminex bead‐based ELISA kit (*n* = 3). Statistical significance was analyzed by unpaired *t* test. Data are presented as mean ± SD. *P*‐value: *P < 0.05, **P < 0.01, ***P < 0.001, ****P < 0.0001.

The oncolytic potential of MNs (TDEVs@OVs) was explored in a xenograft tumor model using C57BL/6 mice carrying TC‐1‐hCD46 tumor. Tumor‐bearing mice were categorized into five groups, each receiving specific treatments via intratumoral injections: PBS, MNs (TC‐1), MNs (OVs), OVs, and MNs (TDEVs@OVs) (Figure 4a). As depicted in Figure 4b, treatment with MNs (TC‐1) did not exhibit a notable inhibition of tumor growth. While animals treated with MNs (OVs) and OVs exhibited moderate tumor growth suppression, the significant reduction in TC‐1 tumor growth and prolongation of survival were observed in the MNs (TDEVs@OVs)‐treated group (Figure 4c–4f and Figure S17). No significant alterations were observed in the tumor‐bearing mice's body weights or histopathological examination of the major organs (Figures S18–S19). Moreover, hepatic and renal functional indicators, such as alanine aminotransferase, aspartate aminotransferase, creatinine, and blood urea nitrogen, exhibited no significant alterations (Figure S20). Besides, the long‐term safety profile of MNs (TDEVs@OVs) was evaluated. As shown in Figures S21–S22, C57BL/6 mice exhibited no significant organ damage or abnormal changes in hepatic and renal function indicators after 30 days of drug administration, demonstrating the favorable safety profile of the MNs (TDEVs@OVs). Furthermore, we also determined the levels of various cytokines in the tumor for all the groups. Quantification of IL‐1*β*, IFN‐*γ*, IL‐6, IL‐12P70, TNF‐*α*, IL‐23, and IL‐27 demonstrated that all tested cytokine levels increased in the MNs (TDEVs@OVs)‐treated group (Figures 4g and S23). As some of the cytokines were mainly secreted by DCs, such as IL‐6, IL‐12P70, TNF‐*α*, IL‐23, and IL‐27, further confirming the effective prevention of DCs exhaustion and the promotion of adaptive immune responses after the MN‐based *in situ* TDEVs‐cloaked OVs therapy.

**FIGURE 5:**
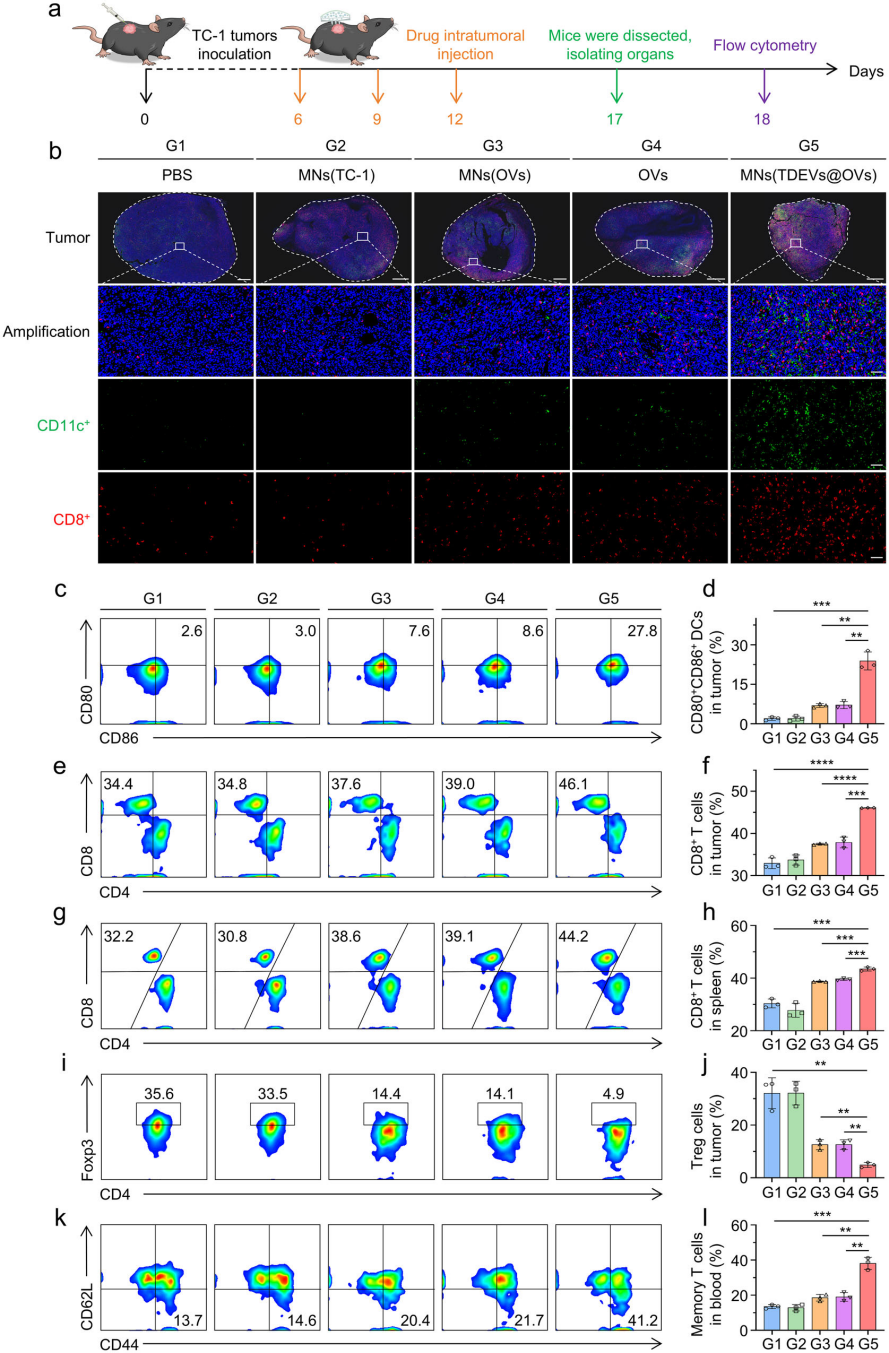
***In vivo* immunoactivation capacity of the MN‐based *in situ* TDEVs‐cloaked OVs**. (a) Schematic illustration of the immunology assessment experiments of the MN using TC‐1‐hCD46 xenograft tumor‐bearing C57BL/6 female mice model. (b) The immunofluorescence images of CD11c^+^ DC cells and CD8^+^ T cells in tumors. Scale bars: 1 mm (Tumor), 50 µm (Amplification). (c, d) Representative flow cytometric evolution images as well as relative quantification of DC cells (CD45^+^CD11c^+^CD80^+^CD86^+^) in the tumor. (e, f) Representative flow cytometric evolution images as well as relative quantification of CD8^+^ T cells (CD45^+^CD3^+^CD8^+^) in the tumor. (g, h) Representative flow cytometric evolution images as well as relative quantification of CD8^+^ T cells (CD45^+^CD3^+^CD8^+^) in the spleen. (i, j) Representative flow cytometric evolution images as well as relative quantification of Treg cells (CD45^+^CD3^+^CD4^+^FOXP3^+^) in the tumor. (k, l) Representative flow cytometric evolution images as well as relative quantification of effector memory T cells (CD45^+^CD3^+^CD8^+^CD62L^−^CD44^+^) in the blood. Statistical significance was analyzed by unpaired *t* test. Data are presented as mean ± SD. (*n* = 3). *P*‐value: *P < 0.05, **P < 0.01, ***P < 0.001, ****P < 0.0001.

DCs play a crucial role in connecting tumor antigens with T cell responses. Mature DCs express significant levels of co‐stimulatory molecules like CD80 and CD86, which can facilitate T cell proliferation, differentiation, and survival. Herein, immunofluorescence was utilized to explore the immunostimulatory potential of the MN in the TC‐1‐hCD46 tumor‐bearing C57BL/6 mice model in Figure 5a. The tumor samples were labeled with fluorescence‐tagged antibodies to evaluate the infiltration of CD11c^+^ DC cells and CD8^+^ T cells. As depicted in Figure 5b, a notable influx of CD11c^+^ DC cells and CD8^+^ T cells was observed in tumors treated with MNs (TDEVs@OVs) in comparison to the control groups. Moreover, mature DCs (CD45^+^CD11c^+^CD80^+^CD86^+^) within the tumors were analyzed by flow cytometry after the third treatment, revealing a higher proportion in the MNs (TDEVs@OVs)‐treated group, indicative of enhanced recruitment and maturation of DCs crucial for activating tumor‐specific T cells (Figure 5c–5d and Figure S24). Besides, flow cytometry analysis of cytotoxic T lymphocytes (CD45^+^CD3^+^CD8^+^) in tumors and spleen revealed a heightened presence of CD8^+^ T cells in the MNs (TDEVs@OVs) group, resulting in bolstered antitumor responses (Figure 5e–5h and Figures S25–26). The notable reduction in regulatory T cells (CD45^+^CD3^+^CD4^+^FOXP3^+^) was observed within the group treated with MNs (TDEVs@OVs), indicating a reshaping of immunosuppressive conditions (Figure 5i–j and Figure S27). Further analysis involved the collection and examination of memory T cells in the blood (CD45^+^CD3^+^CD8^+^CD62L^−^CD44^+^) to assess the MNs (TDEVs@OVs)‐triggered antitumor immune memory effect (Figure 5k–5l and Figure S28). Results demonstrated an elevated presence of memory T cells in the MNs (TDEVs@OVs) group, showcasing the potent immune memory protection induced by MNs (TDEVs@OVs) as an effective approach for sustained long‐term immune responses.

### 2.4 *In Vivo* Tumor Recurrence and Metastasis Suppression

**FIGURE 6:**
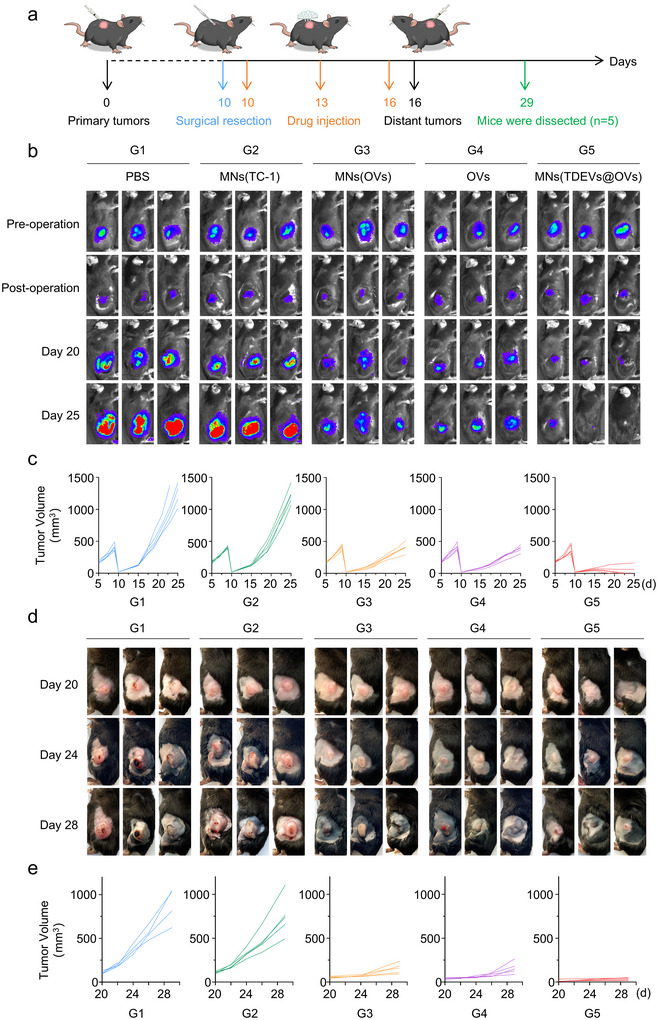
***In vivo* the capacity of the MN‐based *in situ* TDEVs‐cloaked OVs for inhibiting tumor recurrence and metastasis**. (a) Schematic illustration of the inhibiting tumor recurrence and metastasis investigation of the MNs(TDEVs@OVs) on TC‐1‐hCD46 xenograft tumor‐bearing C57BL/6 female mice model. (b) Bioluminescence images of primary tumor surgery and postoperative recurrence. (c) Individual primary tumor growth kinetics in different groups (*n* = 5). (d) Representative photos of distant tumors metastasis. (e) Individual distant tumor metastasis kinetics in different groups (*n* = 5).

Upon the basis of the above immunocyte‐mediated antitumor immune response, we further investigated the therapeutical effects of the MNs (TDEVs@OVs) on residual tumors after surgery (Figure 6a). Female C57BL/6 mice bearing TC‐1‐hCD46 xenograft tumors were segregated into five groups. Following the primary tumor volume reaching 400 mm^3^, excision of the tumors was conducted, leaving 5% of residual tumor mass to assess the recurrence of the residual primary tumor post‐surgery, which was monitored using IVIS imaging and vernier calipers. Although animals treated with MNs(OVs) and OVs could moderately inhibit tumor recurrence, the MNs (TDEVs@OVs)‐treated group showed the optimal capability in suppressing tumor recurrence. Three out of five mice in this group showed undetectable tumors, while the other two mice showed weak tumor recurrence (Figure 6b–6c and Figures S29–S30). Moreover, distant tumors were inoculated after 16 days of therapy to mimic tumor metastasis. The results revealed that the group treated with MNs (TDEVs@OVs) exhibited the most potent anti‐metastatic efficacy (Figure 6d–6e and Figures S31–S32). Additionally, no notable fluctuations in body weight were observed over the course of 29 days (Figure S33). Collectively, the employment of the MNs (TDEVs@OVs) exhibited both high therapeutic effectiveness and favorable biosafety profiles.

## 3 Discussion

In this study, we describe a platform based on the MN for *in situ* secretion of TDEVs‐cloaked OVs. The strategy of loading TC‐1 cells pre‐infected with OVs into the upper reservoir of hollow MN could prevent the passage of tumor cells while facilitating a continuous release of TDEVs@OVs. The released TDEVs@OVs exhibited the ability to specifically target cancer cells at tumor sites. More importantly, they were also able to reduce uptake by DCs through the signaling mechanism of “Don't eat me.” The selective cytotoxicity against tumor cells within the tumor microenvironment efficiently prevents DCs exhaustion to enhance DC‐mediated T cell immunity and maximize the immunostimulatory potential of OVs. In addition, the MN array destroyed the inherent physical barriers and facilitated uniform drug distribution within the tumor region, further augmenting the antitumor efficacy of the MNs (TDEVs@OVs).

Despite the promising results, several aspects concerning safety, generalizability, and clinical translation warrant further discussion. Firstly, the incorporation of live tumor cells into a medical device raises legitimate safety concerns, including potential leakage of viable tumor cells and the theoretical risk of secondary tumor formation. Although our current design incorporates a polycarbonate membrane that effectively retains cells while permitting nanovesicle passage, rigorous long‐term safety assessments (including tests for device integrity under physiological conditions and tumorigenicity studies in immunocompetent and immunocompromised models) will be essential before clinical application. Future iterations could also explore the use of irradiated or replication‐incompetent tumor cells to further mitigate any residual risks.

Secondly, while our platform showed significant efficacy in the TC‐1‐hCD46 xenograft model, its generalizability across other tumor types and animal species remains to be established. Different cancers exhibit heterogeneous expression of surface markers, which may influence OV tropism and TDEV homing. Further studies employing a panel of cancer cell lines and patient‐derived xenografts, as well as testing in larger animal models (e.g., rabbits or pigs), will help evaluate the broader applicability of this approach. In addition, the versatility of the platform for delivering other therapeutic cargoes (such as cytokines, siRNA, or chemotherapeutics) via different extracellular vesicles merits exploration.

Thirdly, regarding clinical translation, our system must be evaluated against existing standard therapies in well‐controlled comparative studies, such as intratumoral OV injection. Although our MN platform offers advantages in terms of sustained release and improved distribution, scalability, manufacturing reproducibility, and sterility assurance present significant challenges. Establishing robust quality control standards for the production of TDEVs@OVs will be critical. Moreover, patient‐specific factors such as tumor accessibility, skin thickness, and immune status may influence treatment outcomes and require careful consideration in clinical trial design.

In summary, the MN‐based *in situ* TDEV‐cloaked OV platform represents a novel strategy to enhance oncolytic virotherapy by minimizing off‐target immune cell toxicity and promoting adaptive antitumor immunity. While the current proof‐of‐concept data are encouraging, future work should focus on addressing the safety, generalizability, and manufacturability challenges outlined above. With continued optimization and validation, this approach holds considerable promise for augmenting cancer immunotherapy.

## 4 Materials and Methods

### 4.1 Materials

Oncolytic adenovirus 11‐Tel (OVs) expresses a green fluorescent protein (GFP) under the control of the telomerase reverse transcriptase (TERT) promoter and Ad5 enhancer, which was extensively supplied by Beijing Bio‐Targeting Therapeutics Technology Co., Ltd. and Zhengzhou University. The engineered non‐small cell lung (TC‐1) cancer cell line expressing the human CD46 receptor was supplied by the Sino‐British Research Centre of Zhengzhou University for this collaboration.

### 4.2 Fabrication and Characterization of the MN

The MN array base, featuring 9 channels for securing hollow MN, and the holder of the MN were designed using SolidWorks 2022. Employing a high‐resolution micromachining process (precision ranging from 0.01 to 0.04 mm), the base and holder of patches. The stainless‐steel hollow MN was designed and cut with approximately 1450 µm in height, 310 µm in base diameter, and 160 µm in inner diameter. Subsequently, these needles were assembled into the base to form an array, and epoxy resin was applied to firmly secure the MN in place. The customized polycarbonate membrane with a 3 µm aperture was provided by the LABSELECT (China) company, while the latex film was precisely cut into the desired shape using customized punches. Upon completion of the individual subcomponents, the integrated process followed the assembly process diagram. The connection between the different units was secured using resin or glue. Finally, a biological double‐sided adhesive was affixed to the bottom of the patch to ensure optimal adhesion to the skin.

TC‐1 cells were cultured in DMEM medium supplemented with 10% FBS, penicillin (100 U/mL), and streptomycin (100 µg/mL). The cells were incubated under 5% CO_2_ at 37°C. Briefly, 10^7^ TC‐1 cells were infected with 10^9^ pfu OVs at an MOI of 100 pfu/cell. After incubation for 12 h, the cells were washed with PBS and collected, resuspended with serum‐free DMEM, and loaded into the MN. 48 h later, TDEVs@OVs released from the MN were collected for further use. Western blot analysis was conducted to detect exosome markers.

### 4.3 Isolation of TDEVs

TC‐1 cells were cultured in DMEM medium until they reached 80% confluence. Subsequently, the medium was replaced with serum‐free culture medium for 48 h before collecting the supernatant for further processing. The TDEVs were firstly isolated utilizing differential ultracentrifugation techniques. In brief, the collected supernatant was centrifuged at 300 × g for 10 min, followed by centrifugation at 2000 × g for 10 min, 10 000 × g for 30 min, and then ultracentrifuged at 100 000 × g for 90 min. Then, the TDEVs were further purified by iodixanol density gradient ultracentrifugation. In brief, a discontinuous iodixanol gradient (Sigma‐Aldrich) was prepared in an ultracentrifuge tube containing 1.5 mL of 40%, 2 mL of 20%, 2 mL of 15%, and 1.5 mL of 10% iodixanol. The TDEVs sample was layered on top of the gradient and centrifuged at 100,000 × g for 18 hours at 4°C using the SW 41Ti rotor (Beckman Coulter). Fractions were then collected from the top, diluted in PBS, and centrifuged at 100,000 g for 90 min to remove the iodixanol. The pellets were resuspended in PBS, and the density of each fraction was measured with an Abbe refractometer. Fractions corresponding to a typical TDEVs density range (1.13‐1.19 g/mL) were pooled and stored at ‐80°C for subsequent functional assays, such as cytotoxicity.

### 4.4 Characterization of the TDEVs@OVs

TDEVs@OVs were diluted with a suitable volume of PBS, deposited on a copper grid, and stained with phosphotungstic acid. The dried grids were examined under a transmission electron microscope at 80 kV. TDEVs@OVs were observed by using the transmission electron microscope (TEM, JEOL, Japan). The size of TDEVs@OVs was detected by using the Zetasizer instrument (Malvern, UK).

### 4.5 Western Blot Analysis

The protein samples were separated by SDS‐PAGE and subsequently transferred onto a polyvinylidene difluoride (PVDF) membrane. Following this, the PVDF membrane was blocked with 5% skim milk for 1 h and then incubated overnight at 4°C with the primary antibody (ABclonal Biotechnology). Following TBST washing, the membrane was exposed to anti‐mouse IgG HRP‐conjugated antibody for 1 h. Target proteins were visualized utilizing an ECL chemiluminescence detection kit (Meilun, China).

### 4.6 Isolation of BMDCs, BMDMs, and CD3+ T Cells

Briefly, bone marrow cells and spleen cells were extracted from the femurs of C57BL/6 mice aged 6 to 8 weeks. BMDCs were obtained from bone marrow cells and cultured for 6 days in a medium supplemented containing 10% FBS, IL‐4 (20 ng/mL), and GM‐CSF (20 ng/mL). BMDMs were isolated from bone marrow cells and cultured for 7 days in a medium containing 10% FBS and M‐CSF (10 ng/mL). CD3^+^ T cells were isolated from spleen cells using the BeaverBeads Mouse CD3^+^ T Cell Isolation Kit (negative selection).

### 4.7 Immunoprecipitation Assay

The adenovirus‐specific anti‐hexon antibody was mixed with OVs, TDEVs@OVs or lysed TDEVs@OVs (10^8^ pfu/mL) and incubated for 2 h at 4°C. For the lysed TDEVs@OVs group, the sample was pre‐treated with 0.5% Triton X‐100 for 15 min on ice to disrupt the TDEVs membrane prior to the addition of the anti‐hexon antibody. Afterward, protein G‐coated agarose beads (Beyotime, China) were added to the mixture and incubated for 1 h. The mixture was finally centrifuged at 6,000 rpm for 1 min, and the supernatant was collected. The amount of ad11 remaining in the supernatant was determined by qPCR assay.

### 4.8 Cell Viability

BMDCs, BMDMs, CD3^+^ T cells, and DC2.4 cells were plated at a density of 10^4^ cells per well in 96‐well plates and incubated overnight. OVs were added at different concentrations and subsequently incubated at 37°C for 48‐72 h. Cell viability was assessed by CCK‐8 assay.

TC‐1 cells and BMDCs were plated at a density of 10^4^ cells per well in 96‐well plates and incubated overnight. PBS, TDEVs, OVs, TDEVs+OVs, and TDEVs@OVs (5×10^6^ pfu OVs) were added and subsequently incubated for 48 h. (The quantities of OVs and TDEVs were quantified by QPCR and BCA protein assay, ensuring equivalent amounts across groups). Cell viability was assessed by CCK‐8 assay. For the blocking experiments, TC‐1 cells and BMDCs were plated at a density of 10^4^ cells per well in 96‐well plates and incubated overnight as described above. TDEVs, OVs, TDEVs+OVs, and TDEVs@OVs (5×10^6^ pfu OVs) were first pre‐incubated with anti‐CD47 antibody for 2 h, followed by washing and ultrafiltration. The collected samples were then added to cells and incubated for 48 h, after which cell viability was assessed by CCK‐8 assay.

TC‐1 cells were infected with OVs at a MOI of 100 pfu/cell. After incubation for 12 h, the cells were washed with PBS and collected, resuspended with serum‐free DMEM and loaded into the MN. 48 h later, TC‐1 cells were collected, and cell viability was evaluated by a LIVE/DEAD Viability/Cytotoxicity Assay Kit.

### 4.9 Cellular Uptake *In Vitro*

TC‐1 cells and BMDCs were plated at a density of 1×10^5^ cells per well in 12‐well plates and incubated overnight. FITC‐labelled naked OVs and TDEVs@OVs (1×10^7^ pfu/well) were added and co‐incubated for 8 h. Subsequently, cells were washed with PBS and stained with DAPI for 0.5 h. Then, the cellular uptake was observed by CLSM. For the blocking experiments, OVs and TDEVs@OVs were first pre‐incubated with anti‐CD47 antibody for 2 h, followed by washing and ultrafiltration. All other procedures, including cell seeding and drug administration, were performed as described above.

BMDCs‐TC‐1 cell co‐culture model: For TC‐1 cells only and BMDCs only groups, cells were seeded in 6‐well plates at a density of 1×10^5^ cells per well and incubated for 24 h. For the TC‐1 cells mixed with the BMDCs group, 1×10^5^ TC‐1 cells and 1×10^5^ BMDCs were mixed and seeded in 6‐well plates. Then, PBS, FITC‐labelled TDEVs@OVs, and naked OVs (1×10^7^ pfu/well) were added, and after incubation for 8 h, the cells were collected for flow cytometry analysis.

### 4.10 Evaluation of the Immune Activation Ability of TDEVs@OVs in a Co‐Culture System *In Vitro*

1×10^5^ TC‐1 cells were mixed with BMDCs in a 1:1 ratio and incubated overnight. PBS, OVs, and TDEVs@OVs (10^7^ pfu/well) were added and incubated at 37°C. 24 h after pretreatment, CD3^+^ T cells isolated by the CD3^+^ T Cell Isolation Kit were added, and co‐culture was sustained for another 48 h before cell collection for flow cytometry analysis.

### 4.11 Evaluation of the Antitumor Effect of MNs(TDEVs@OVs) *In Vivo*

Female C57BL/6 mice were obtained from the Laboratory Animal Center of Shenyang Pharmaceutical University. All animal procedures adhered to the Guidelines for the Care and Use of Laboratory Animals endorsed by the Institutional Animal Ethical Care Committee (IAEC) of Shenyang Pharmaceutical University.

10^6^ TC‐1 cells were subcutaneously injected into the dorsum of C57BL/6 mice, and mice were randomly assigned into five treatment groups (*n* = 5) using a computer‐generated random number sequence. This ensured an unbiased distribution of initial tumor sizes across all groups before the commencement of treatments. When the tumor reached 150 mm^3^ in size, the mice were injected intratumorally with PBS, MNs(TC‐1), MNs(OVs) (1×10^8^ pfu), OVs (1×10^8^ pfu), and MNs(TDEVs@OVs) (1×10^8^ pfu). The treatments were administered every 3 days for 3 consecutive times, with tumor volume monitored using a vernier caliper. The mice were euthanized through cervical dislocation on the 17th day, and the tumor tissue was isolated and photographed. During the treatment administration and subsequent tumor volume measurements, the investigator performing the injections and measuring tumor dimensions with a caliper was blinded to the group allocations. The treatments were coded by a separate individual not involved in the outcome assessment.

### 4.12 Flow Cytometry Analysis

Tumor tissues, spleens, and peripheral blood were extracted from TC‐1‐bearing C57BL/6 mice. Initially, single‐cell suspensions were prepared from the spleen and tumor tissues. The cells were subsequently labeled with fluorescent antibodies: CD3 (Biolegend, cat. no.100204, 100218), CD4 (Biolegend, cat. no.100432), FOXP3 (Biolegend, cat. no.126404), CD8 (Biolegend, cat. no.100712, 100706), CD44 (Biolegend, cat. no.103008), CD45 (Biolegend, cat. no.103130), CD62L (Biolegend, cat. no.104428), CD80 (Biolegend, cat. no.305205), and CD86 (Biolegend, cat. no.200307), following the manufacturers’ protocols. Flow cytometry was utilized to quantify the percentage of labeled cells, with data analysis conducted using FlowJo software.

It should be noted that conventional flow cytometry has inherent limitations in resolving individual nanoparticles. Signals detected may represent single events or, in some cases, coincident detection of multiple particles or aggregates. Therefore, the data should be interpreted with these technical considerations in mind.

### 4.13 Evaluation of Inhibiting Tumor Recurrence and Metastasis Effect

10^6^ TC‐1 cells were subcutaneously injected into the dorsum of C57BL/6 mice, and mice were randomly assigned into five treatment groups (*n* = 5) using a computer‐generated random number sequence. This ensured an unbiased distribution of initial tumor sizes across all groups before the commencement of treatments. Upon reaching a size of 400‐500 mm^3^, 95% of the primary tumor mass was surgical, the mice received intratumoral administrations of PBS, MNs (TC‐1), MNs (OVs) (1×10^8^ pfu), OVs (1×10^8^ pfu), and MNs (TDEVs@OVs) (1×10^8^ pfu). The treatment regimen was administered every 3 days for 3 consecutive times, during which tumor volumes were assessed using IVIS imaging and a vernier caliper, with the mice weighed every 2 days. Distant tumors were inoculated on the 16th day, and tumor volumes were monitored using a vernier caliper. The mice were euthanized by cervical dislocation on the 29th day, and the tumor tissue was excised and photographed. During the treatment administration and subsequent tumor volume measurements, the investigator performing the injections and measuring tumor dimensions with a caliper was blinded to the group allocations. The treatments were coded by a separate individual not involved in the outcome assessment.

### 4.14 Statistical Analysis

Statistical analyses were conducted using GraphPad Prism 8 software. Data were expressed as mean ± standard deviation and mean ± standard error of the mean. Statistical differences were assessed using Student's *t*‐test, followed by Tukey's post‐hoc analysis. Details regarding sample sizes for each statistical analysis and data preprocessing were provided in the figure legend. Statistical significance was considered at a *P* value below 0.05.

## Author Contributions

**Tianye Wang**: writing – review and editing, writing – original draft, conceptualization. **Sheng Zhao**: methodology. **Zao Ji**: software. **Zhonggui He**: methodology. **Zhenguo Cheng**: methodology. **Zhen Gu**: conceptualization, writing – review and editing. **Yuqi Zhang**: conceptualization, writing – review and editing. **Jin Sun**: writing – review and editing, conceptualization. **Funan Liu**: funding acquisition, writing – review and editing, conceptualization. **Mengchi Sun**: writing – review and editing, conceptualization.

## Conflicts of Interest

Z. G. is the co‐founder of Zenomics Inc., ZCapsule Inc., and µZen Pharma Co., Ltd., and the other authors declare no conflict of interest.

## Supporting information




**Supplementary Materials**: jev270222‐sup‐0001‐Figures.docx

## Data Availability

The data that support the findings of this study are available from the corresponding author upon reasonable request.
