# Supplementary material for: In Situ Sustained Delivery of Tumor Cell‐Derived Extracellular Nanovesicles With Oncolytic Adenoviruses for Potentiating Cancer Immunotherapy
Source: J Extracell Vesicles. 2026 Jan 6;15(1):e70222. doi: 10.1002/jev2.70222 (PMC12775349; doi:10.1002/jev2.70222)
Supplement: Supplementary file 1 — Supplementary Materials: jev270222‐sup‐0001‐Figures.docx [file JEV2-15-e70222-s001.docx]

Supplementary Materials for

***In situ* sustained delivery of tumor cell-derived extracellular nanovesicles with oncolytic adenoviruses for potentiating cancer immunotherapy**

Tianye Wang *et al.*

Corresponding authors: Mengchi Sun, sunmengchi@syphu.edu.cn; Funan Liu, fnliu@cmu.edu.cn; Jin Sun, sunjin@syphu.edu.cn; Yuqi Zhang, yqzhang21@zju.edu.cn.

This PDF file includes:

Fig.S1 to S33


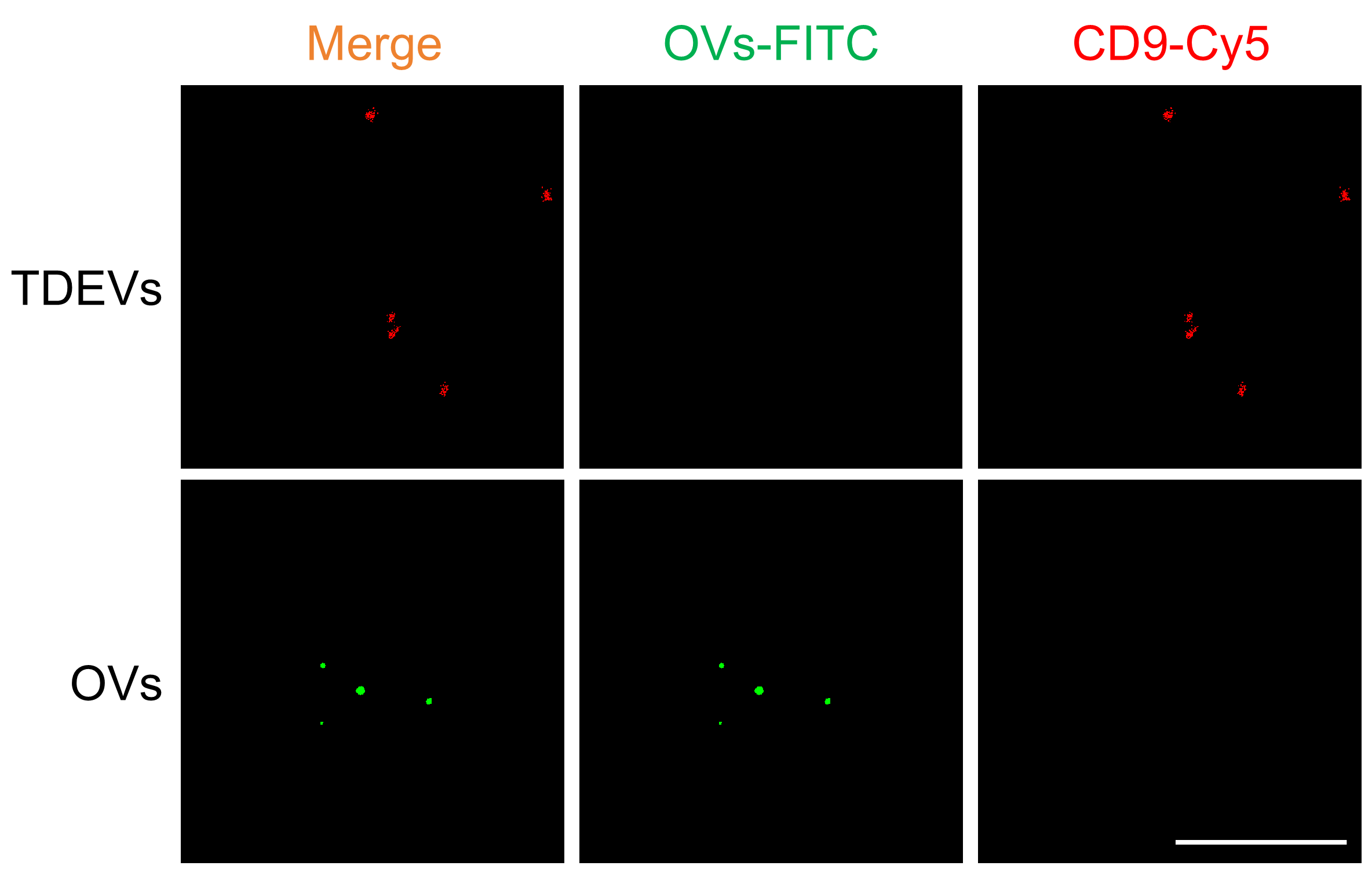


**Fig. S1.** Control groups of the colocalization experiment in **Fig.2i** by CLSM. OVs were labeled with FITC dye (green) while TDEVs was labeled with CD9-Cy5 dye (red); scale bar: 100 μm.


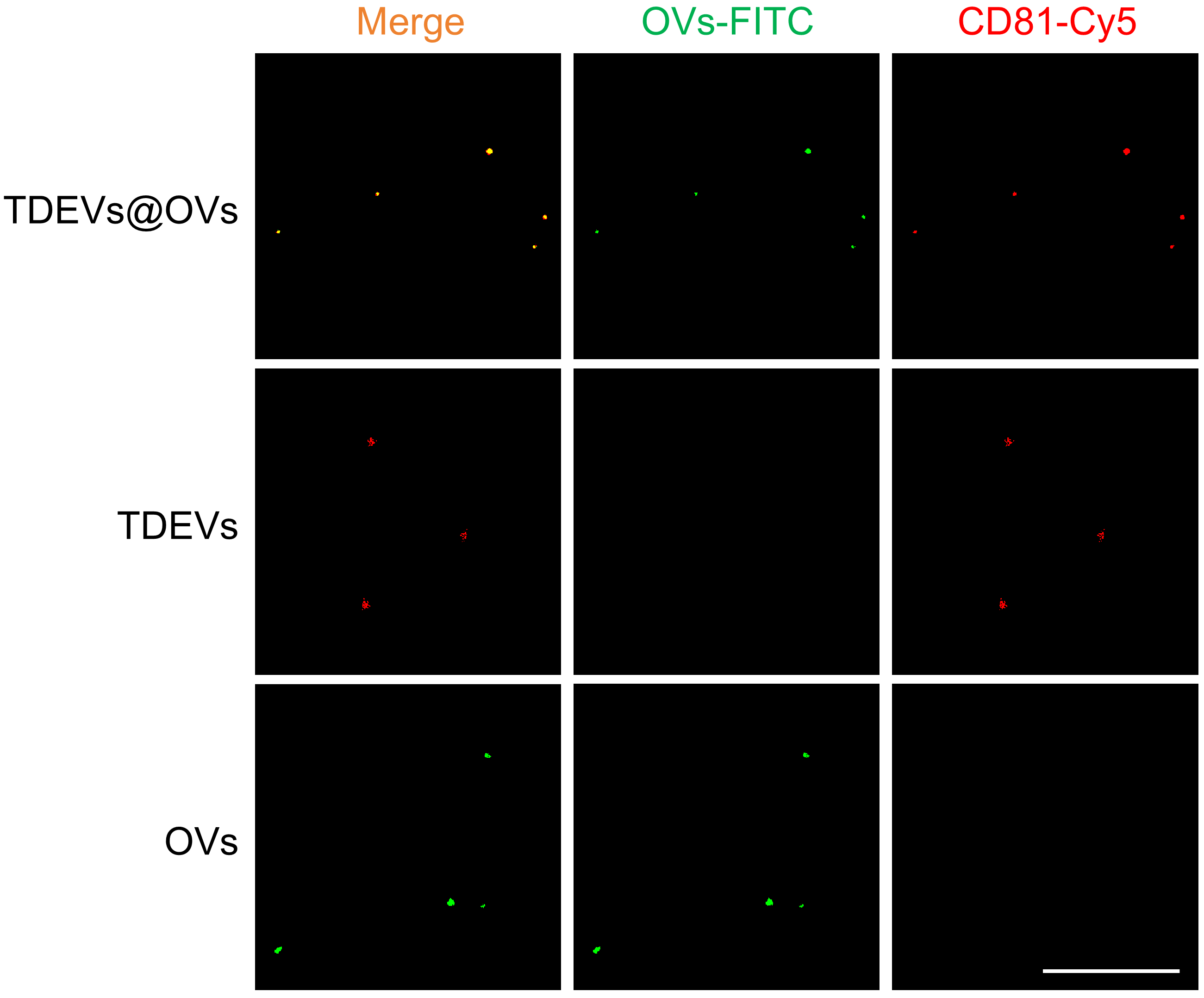


**Fig. S2.** Colocalization of TDEVs@OVs, TDEVs and OVs by CLSM. OVs were labeled with FITC dye (green) while TDEVs was labeled with CD81-Cy5 dye (red); scale bar: 100 μm.


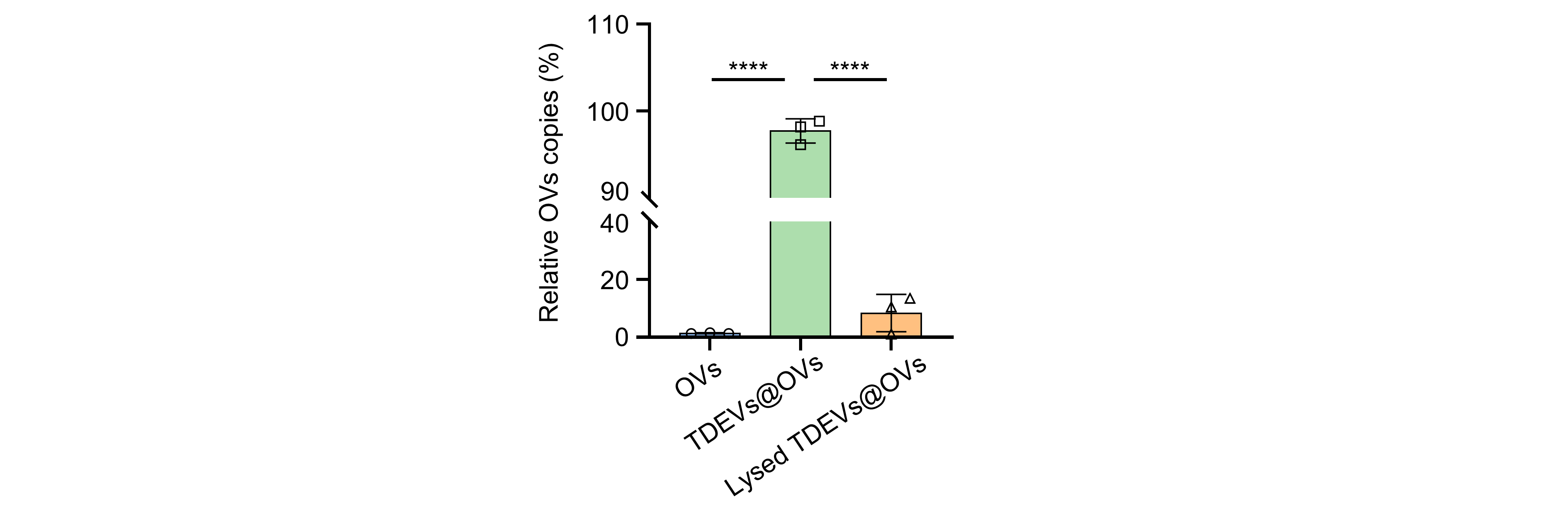


**Fig. S3.** The amount of OVs, TDEVs@OVs and Lysed TDEVs@OVs that escaped from binding with the adenovirus hexon protein antibody in immunoprecipitation assay.


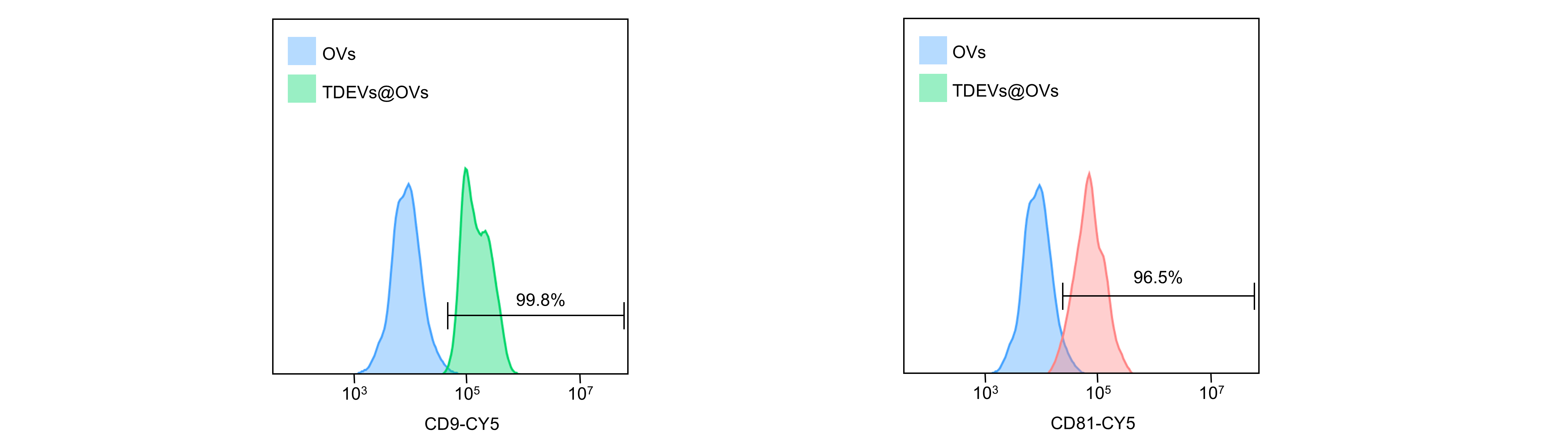


**Fig. S4.** The encapsulation efficiency of TDEVs@OVs evaluated through flow cytometry. TDEVs was labeled with CD9-Cy5.


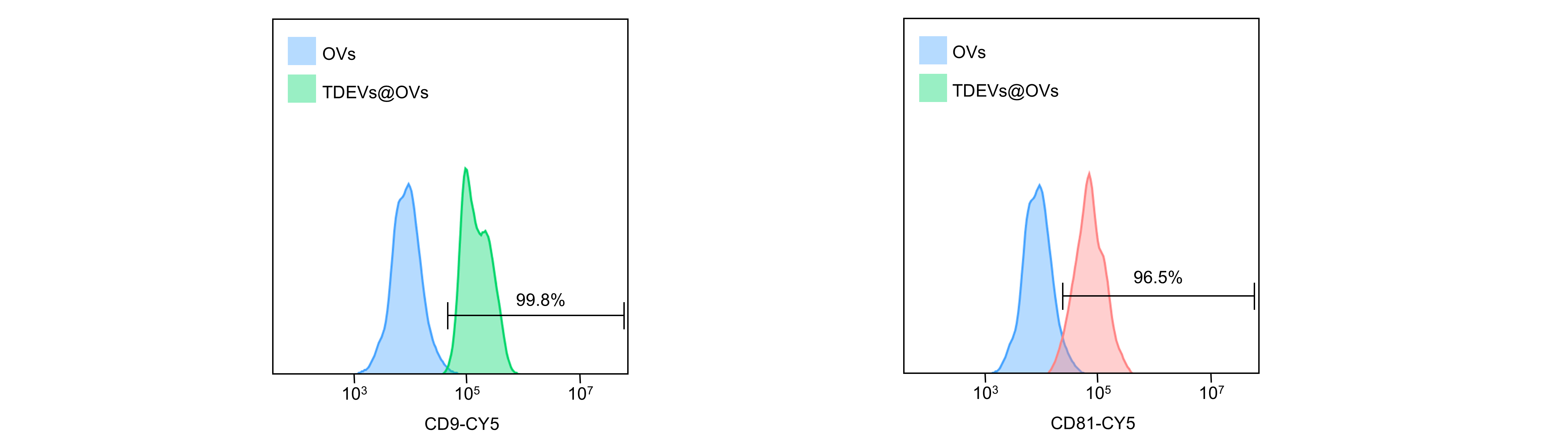


**Fig. S5.** The encapsulation efficiency of TDEVs@OVs evaluated through flow cytometry. TDEVs was labeled with CD81-Cy5.


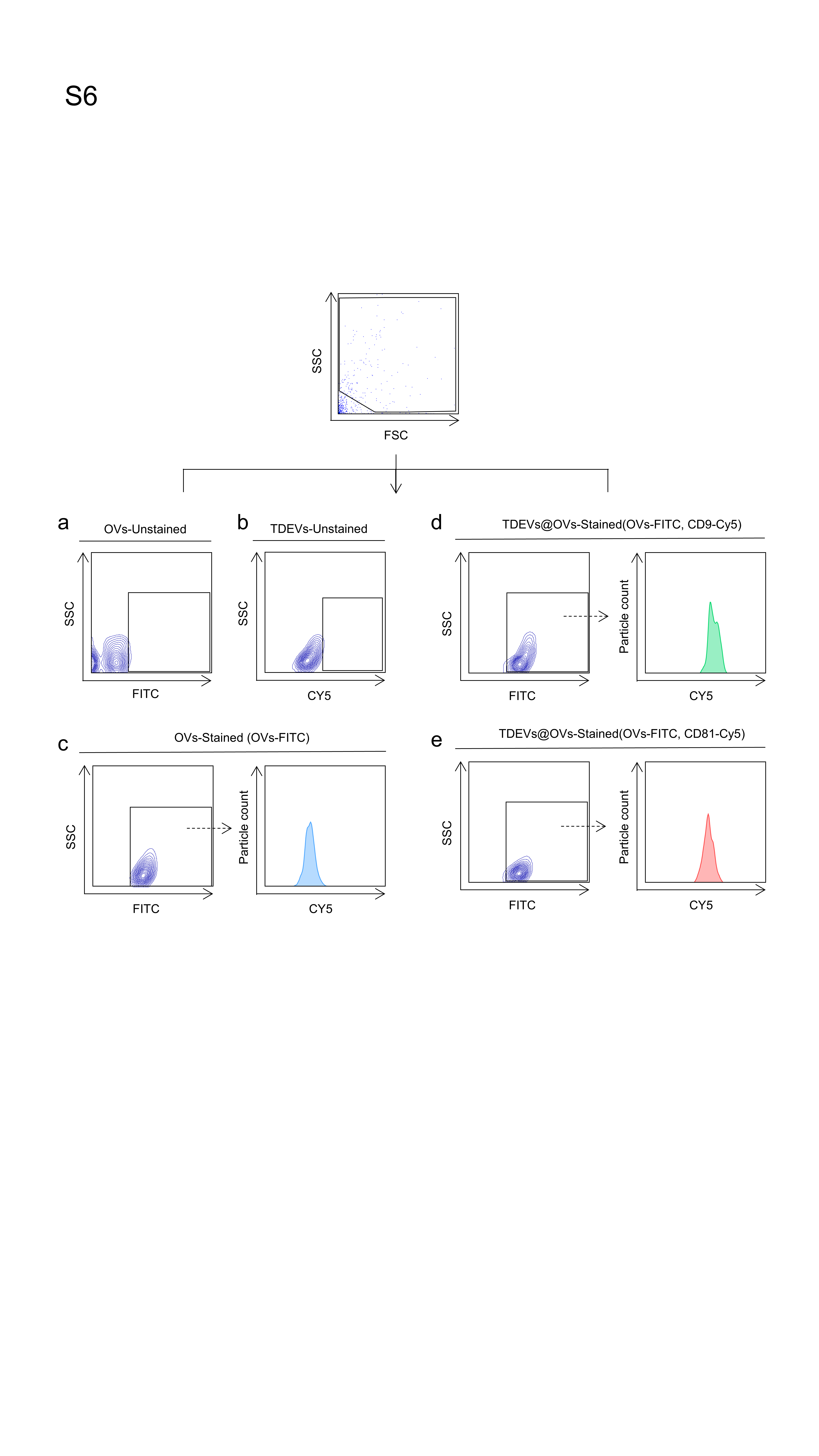


**Fig. S6.** The gating strategy of TDEVs@OVs encapsulation efficiency evaluated through flow cytometry in **Fig.S3** and **Fig.S4**. OVs were labeled with FITC while TDEVs was labeled with CD9-Cy5 or CD81-Cy5.


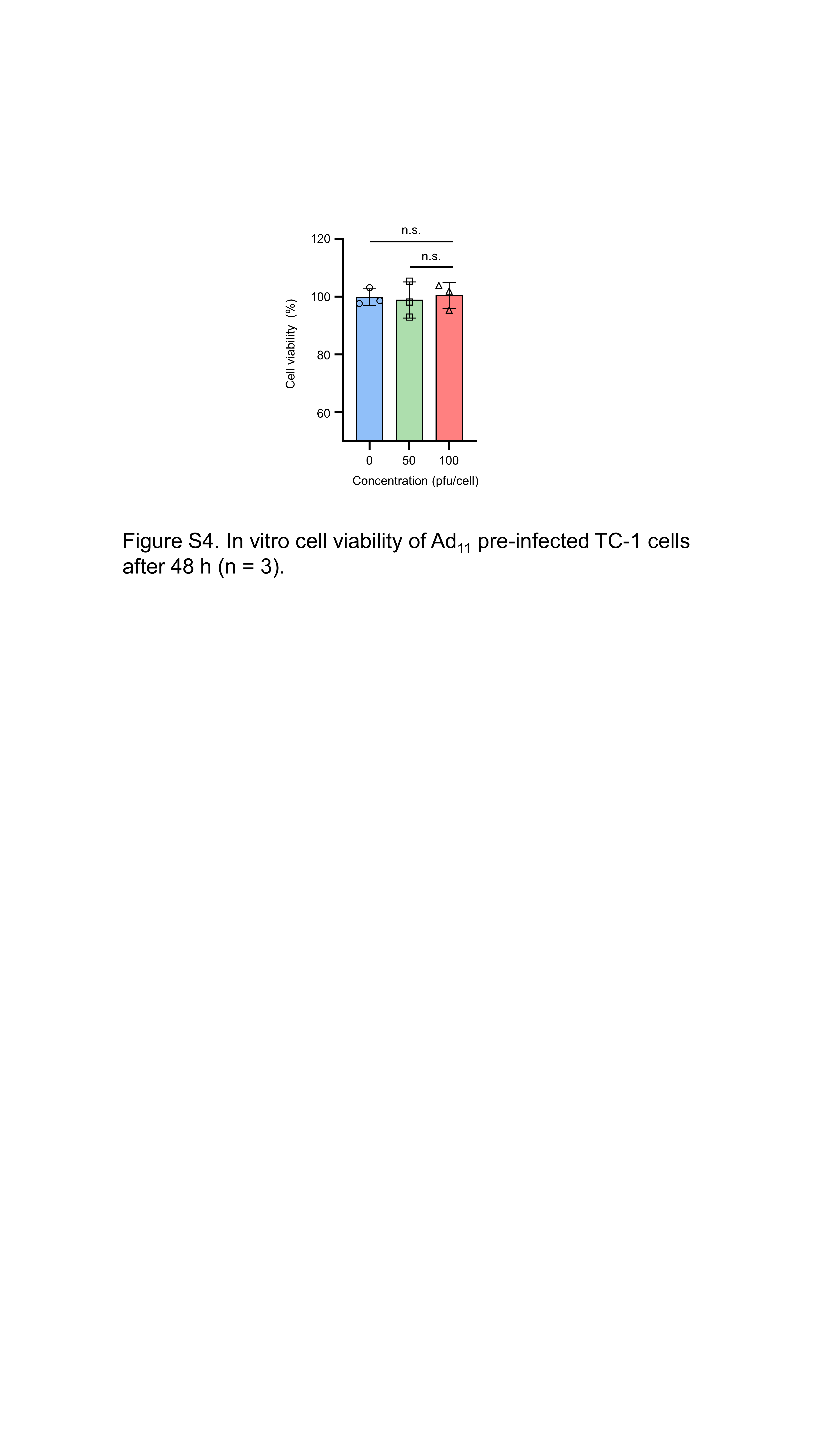


**Fig. S7.** *In vitro* cell viability of OVs pre-infected TC-1 cells after 48 h (*n* = 3). Statistical significance was analyzed by unpaired *t* test.


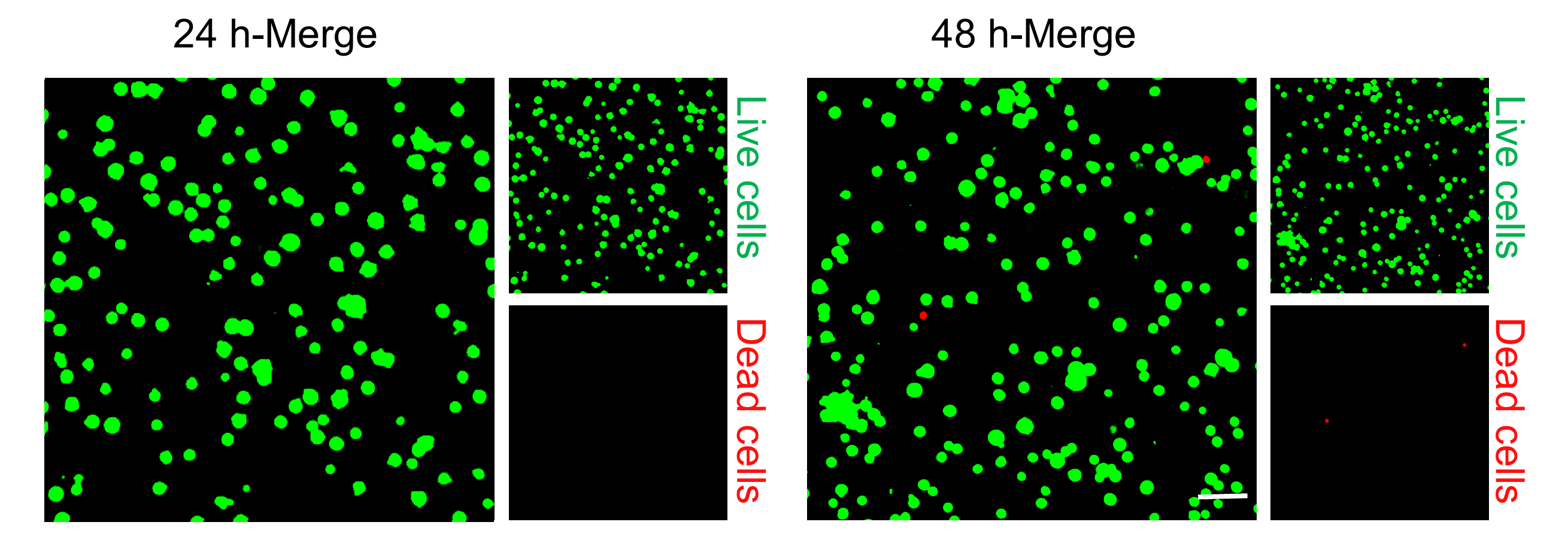


**Fig. S8.** *In vitro* cell viability of OVs pre-infected TC-1 cells within the MN after 48 h by CLSM. Living cells were stained with Calcein (green) and dead cells were stained with PI (red). Scale bar: 100 μm.


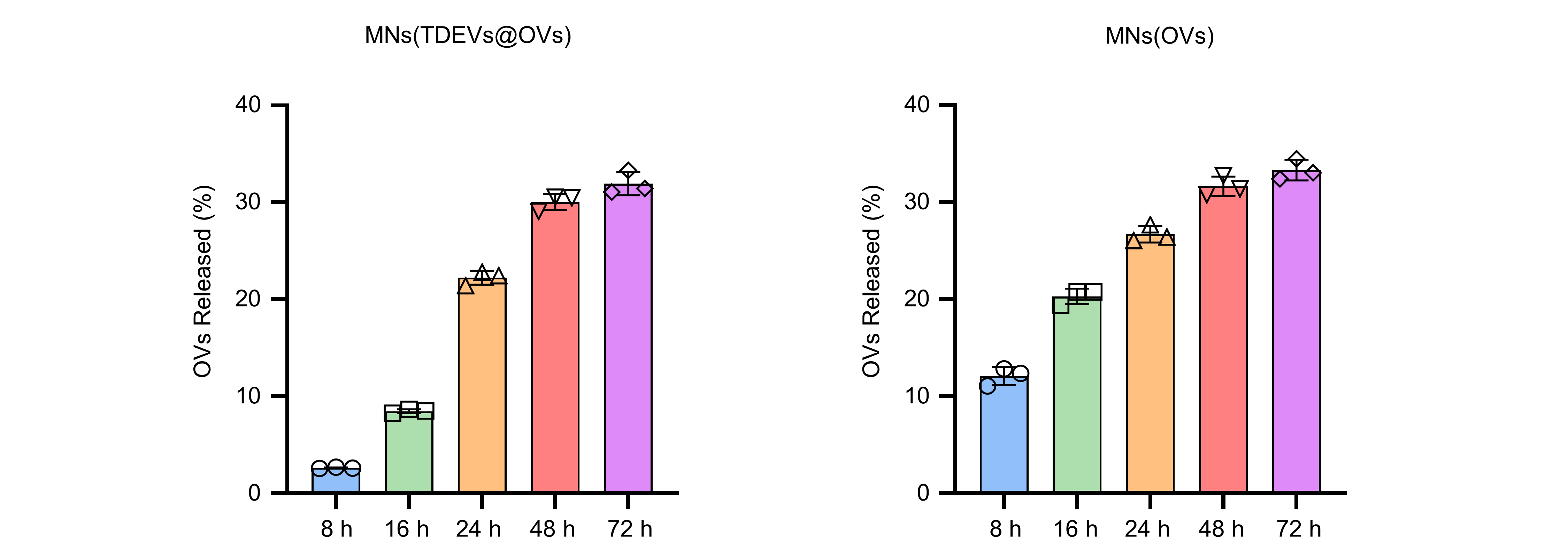


**Fig. S9.** *In vitro* OVs released from the MN (*n* = 3). Data are presented as mean ± SD.


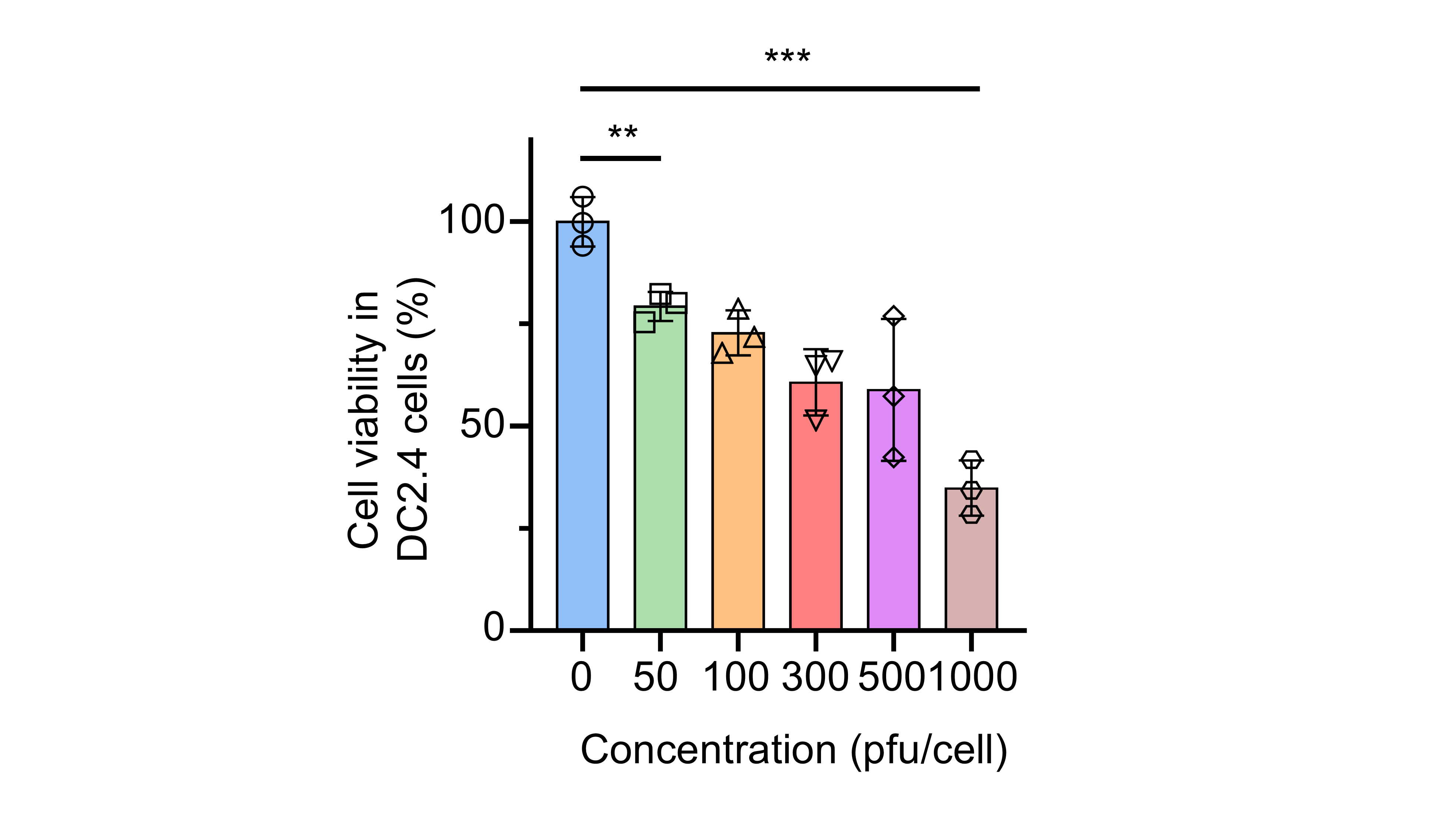


**Fig. S10.** *In vitro* cytotoxicity of different concentration of OVs against DCs (*n* = 3). Statistical significance was analyzed by unpaired *t* test. *P*-value: ***P* < 0.01, ****P* < 0.001.


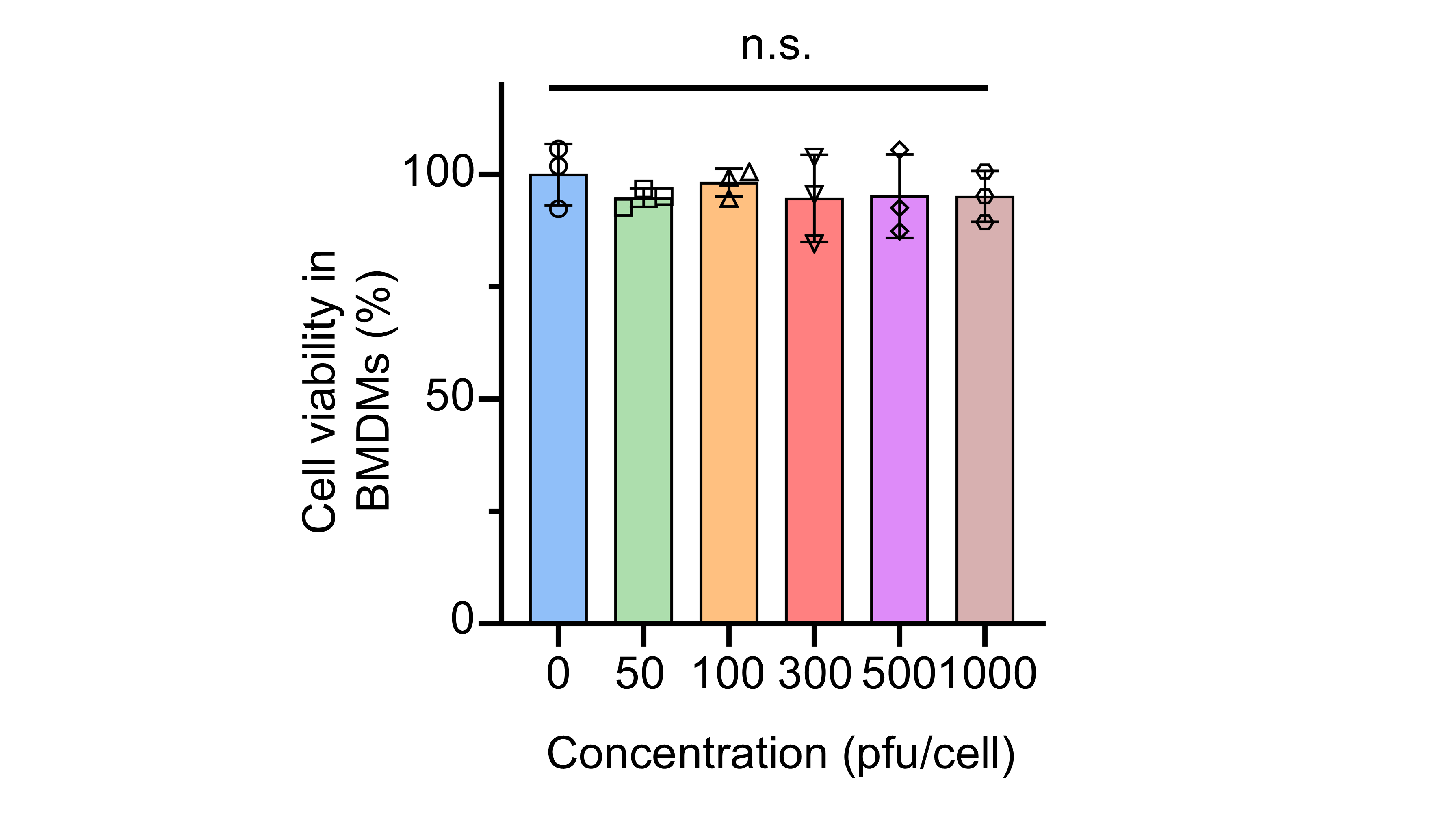


**Fig. S11.** *In vitro* cytotoxicity of different concentration of OVs against BMDM cells (*n* = 3). Statistical significance was analyzed by unpaired *t* test.


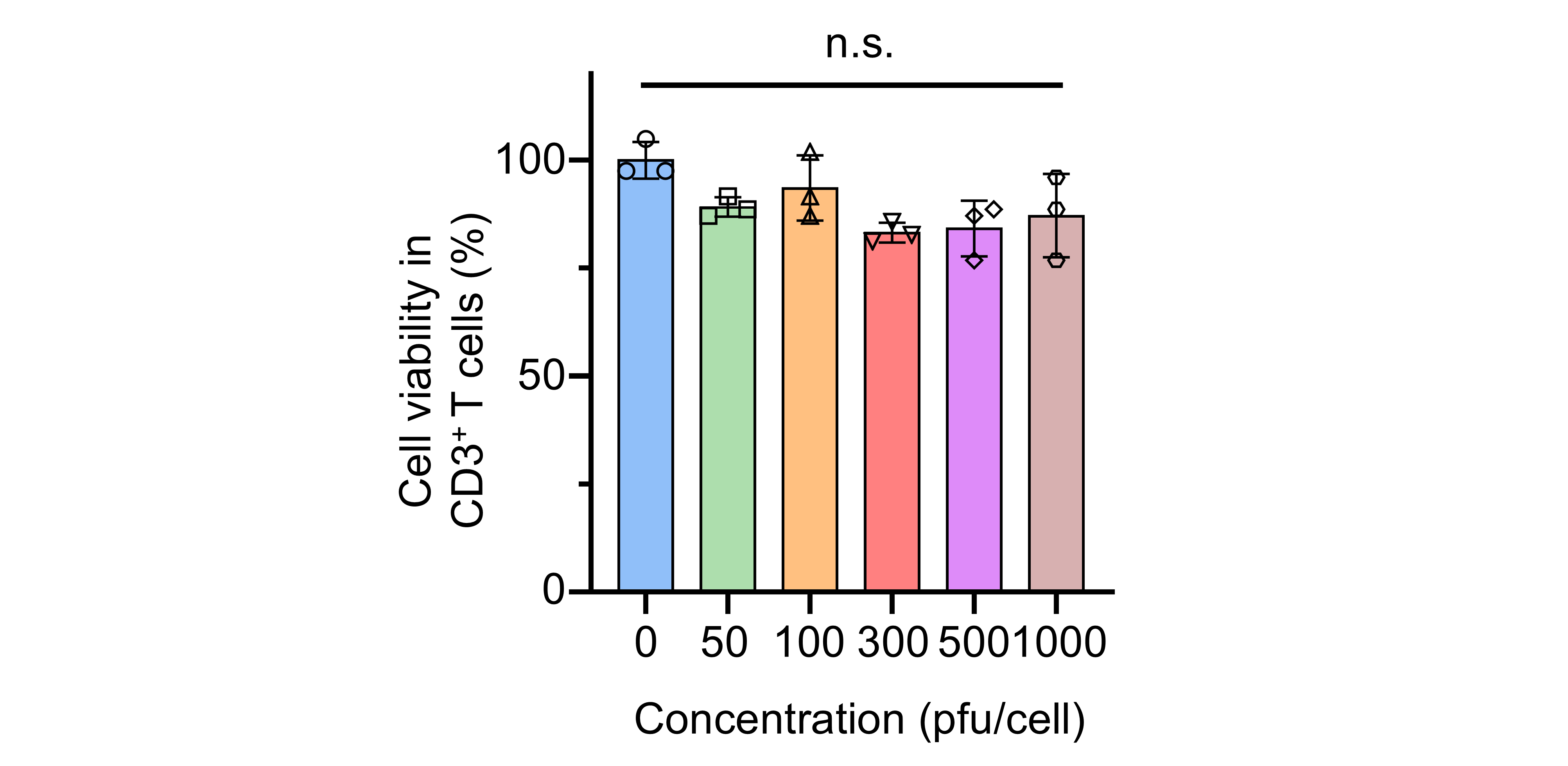


**Fig. S12.** *In vitro* cytotoxicity of different concentration of OVs against spleen-derived CD3^+^ T cells (*n* = 3). Statistical significance was analyzed by unpaired *t* test.
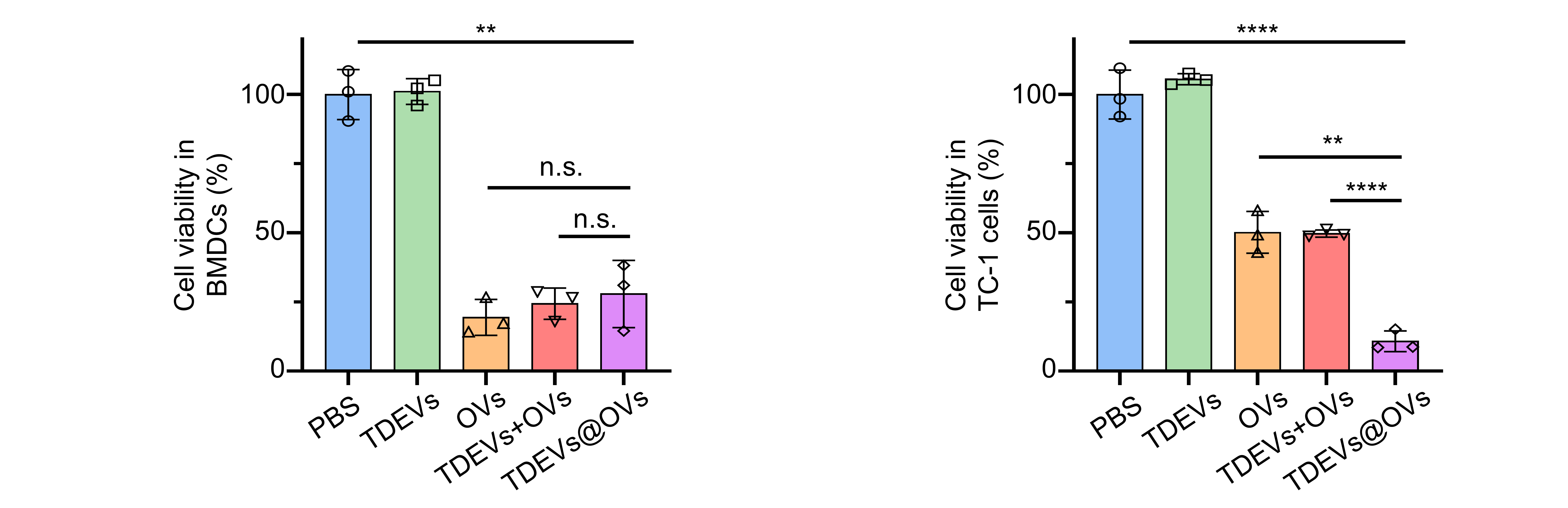


**Fig. S13.** *In vitro* blocking experiments to confirm the cytotoxicity of different samples against BMDCs (left) and TC-1 cells (right) by using anti-CD47 antibody (*n* = 3).


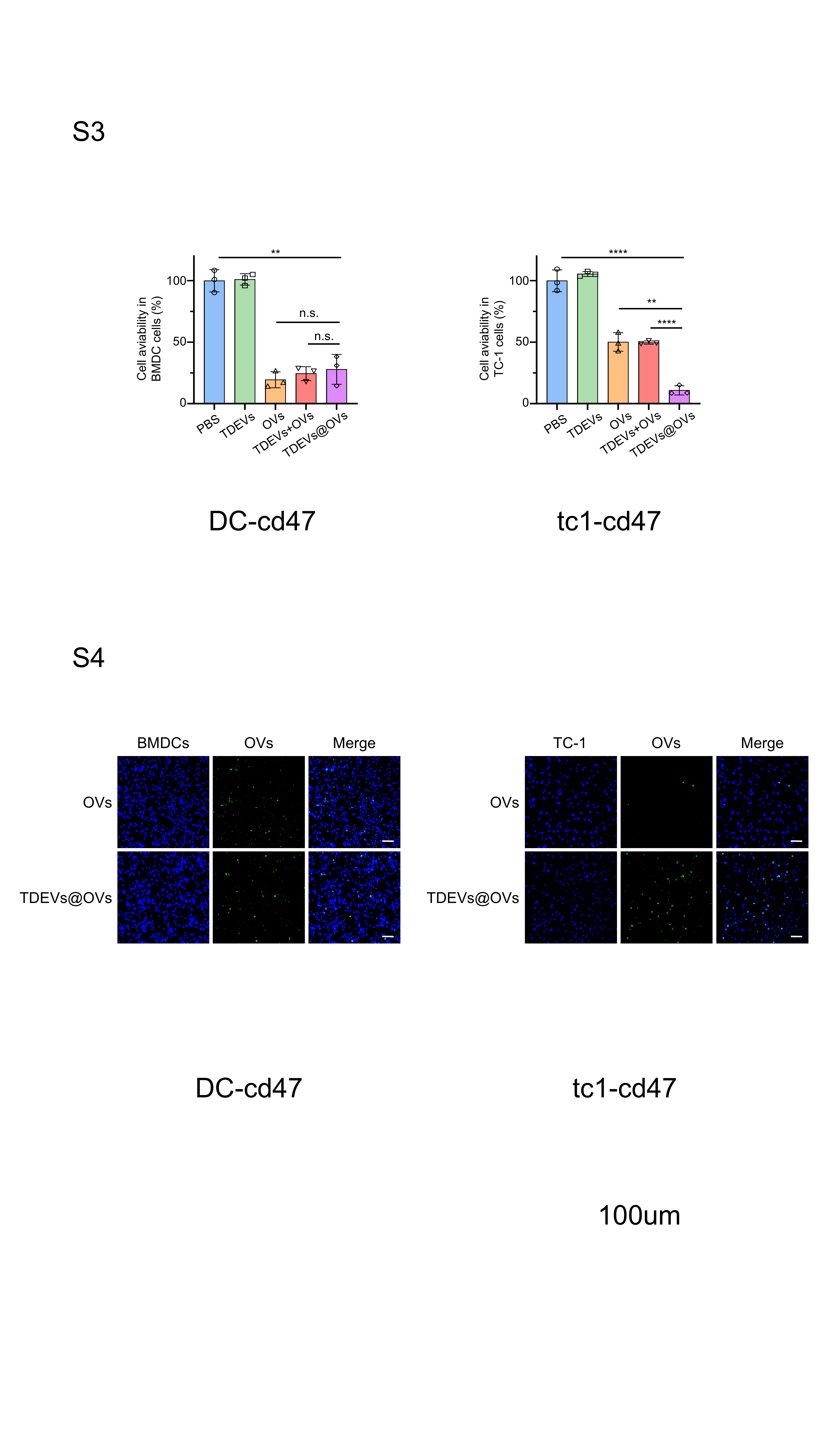


**Fig. S14.** *In vitro* blocking experiments to confirm the FITC-labeled naked OVs and TDEVs@OVs endocytosed by BMDCs (left) and TC-1 cells (right) by using the anti-CD47 antibody. Scale bar: 100 μm.


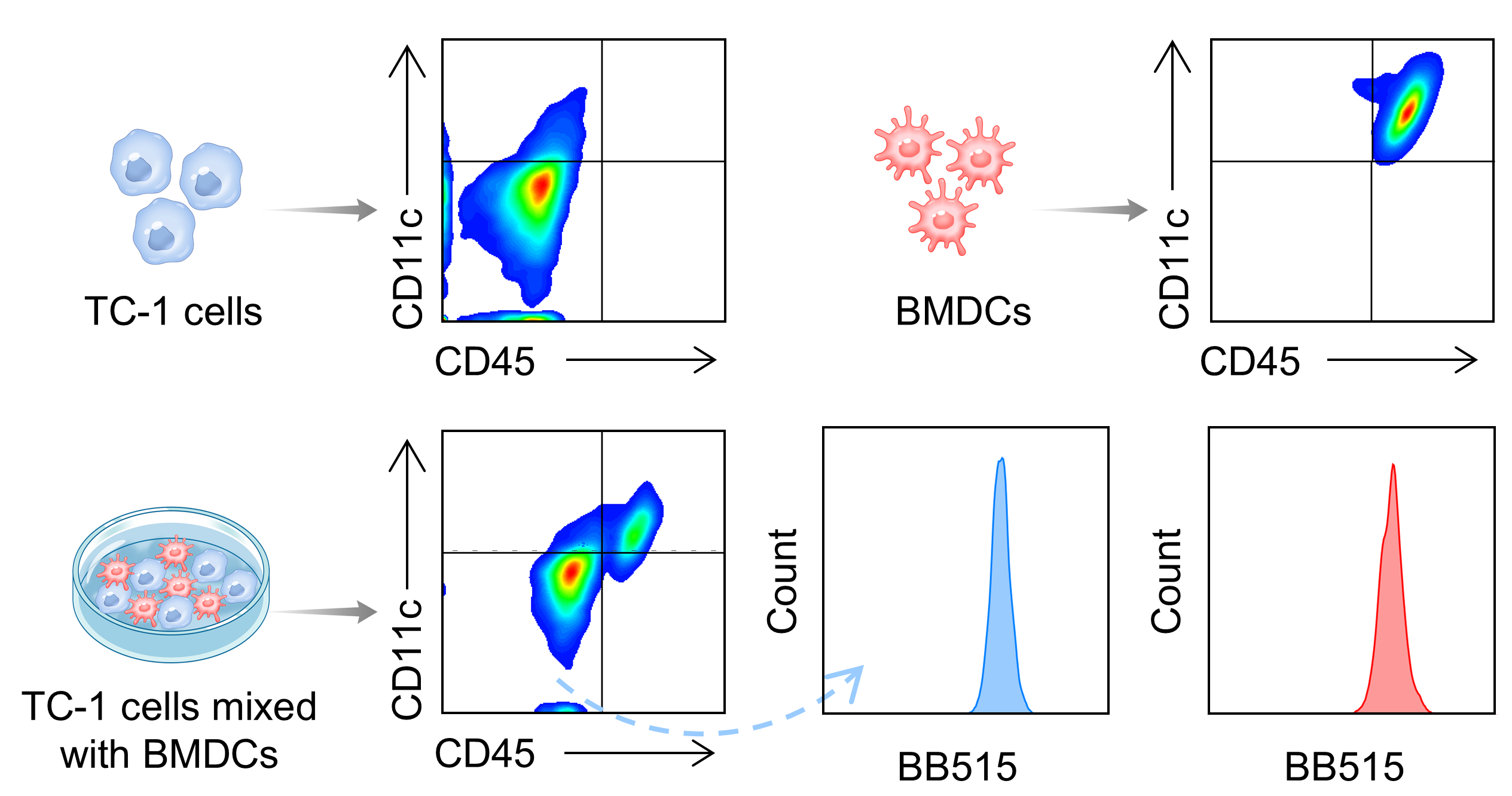


**Fig. S15.** Schematic diagram of the BMDCs-TC-1 cells co-culture model evaluated by flow cytometry.


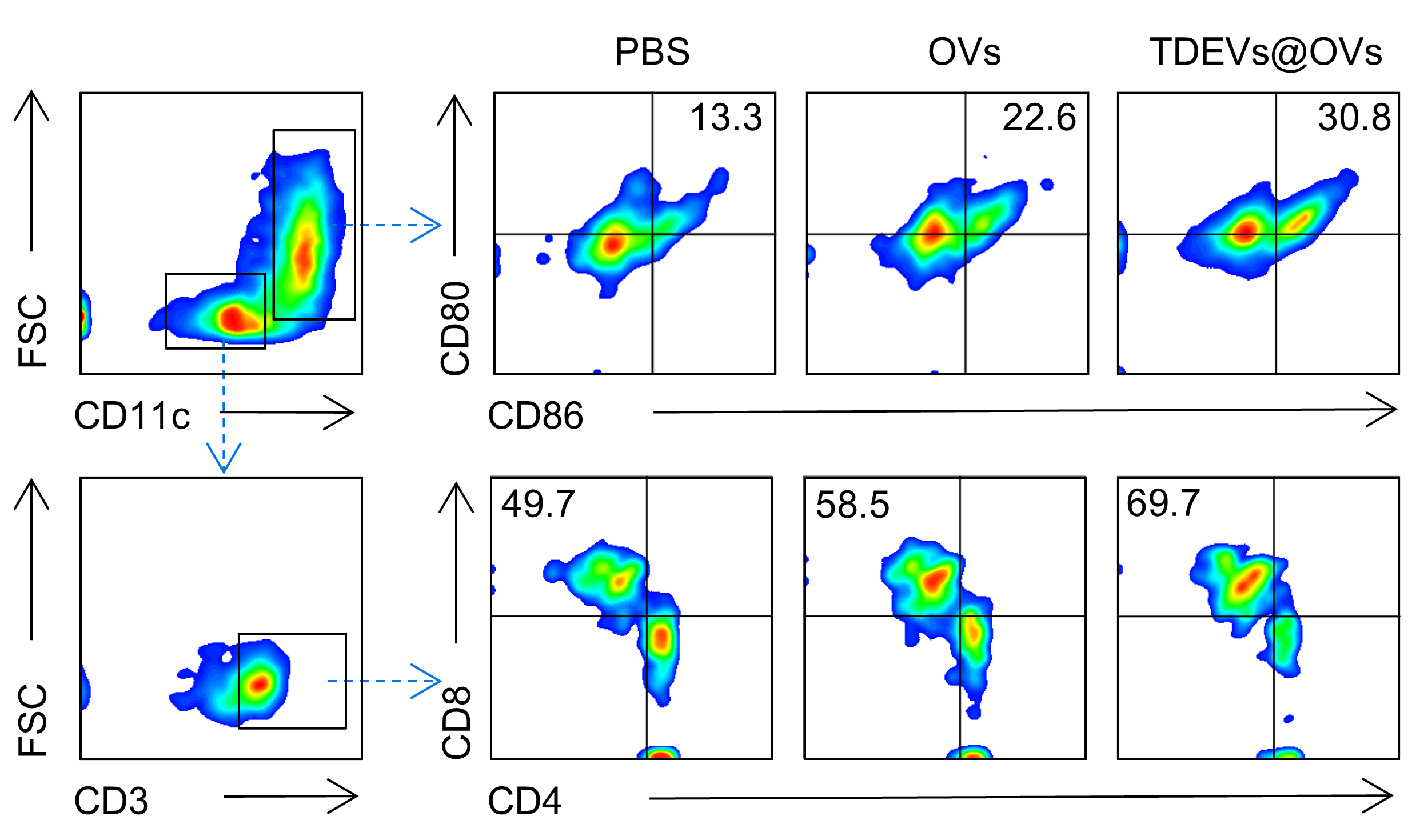


**Fig. S16.** Schematic diagram of the TC-1-BMDCs-CD3^+^ T cells co-culture model evaluated by flow cytometry.


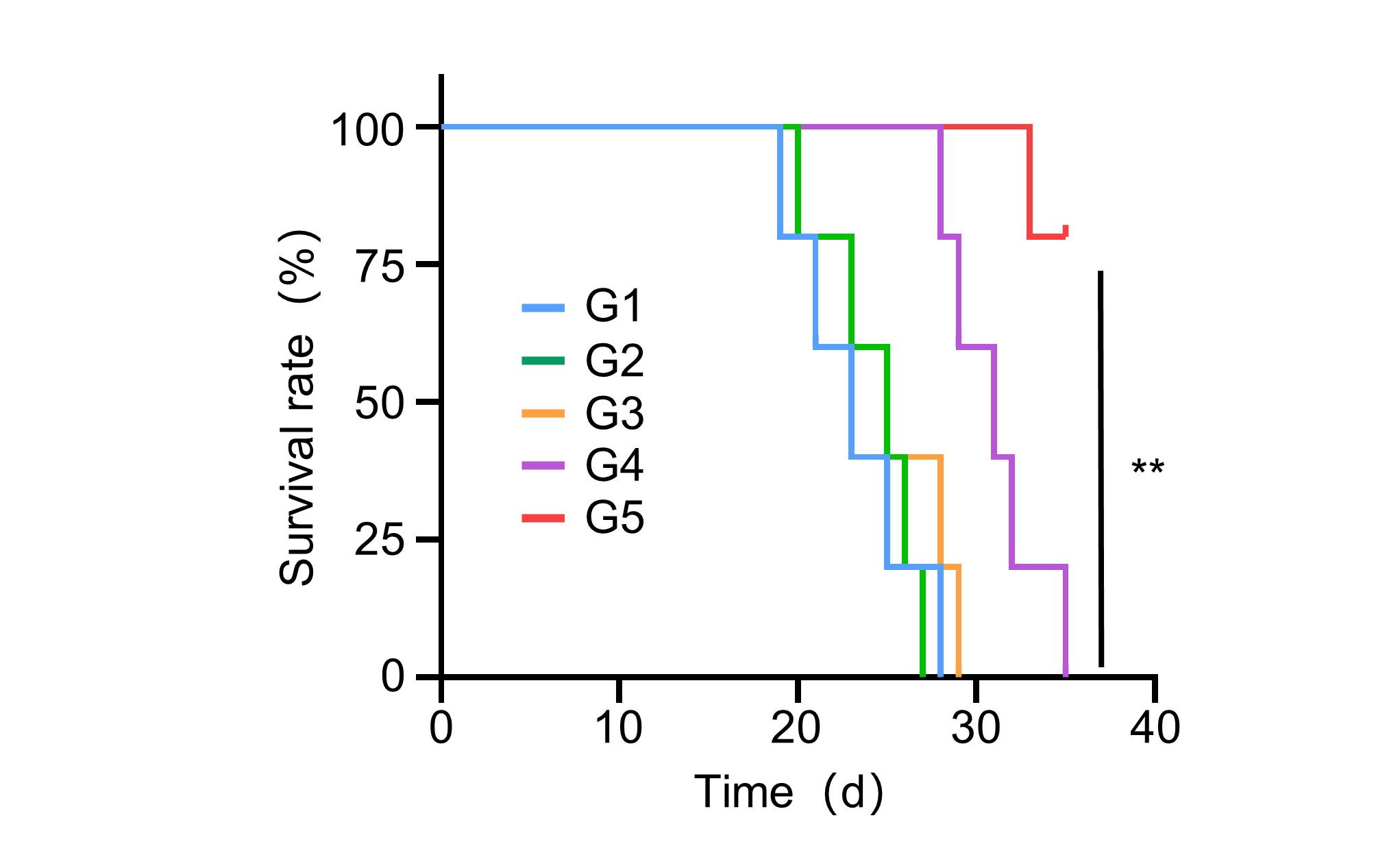


**Fig. S17.** Survival curves of mice receiving each treatment (*n* = 5). Statistical significance was analyzed by two-tailed Student’s t-test. *P*-value: ***P* < 0.01. (G1: PBS, G2: MNs(TC-1), G3: MNs(OVs), G4: OVs and G5: MNs(TDEVs@OVs) ).


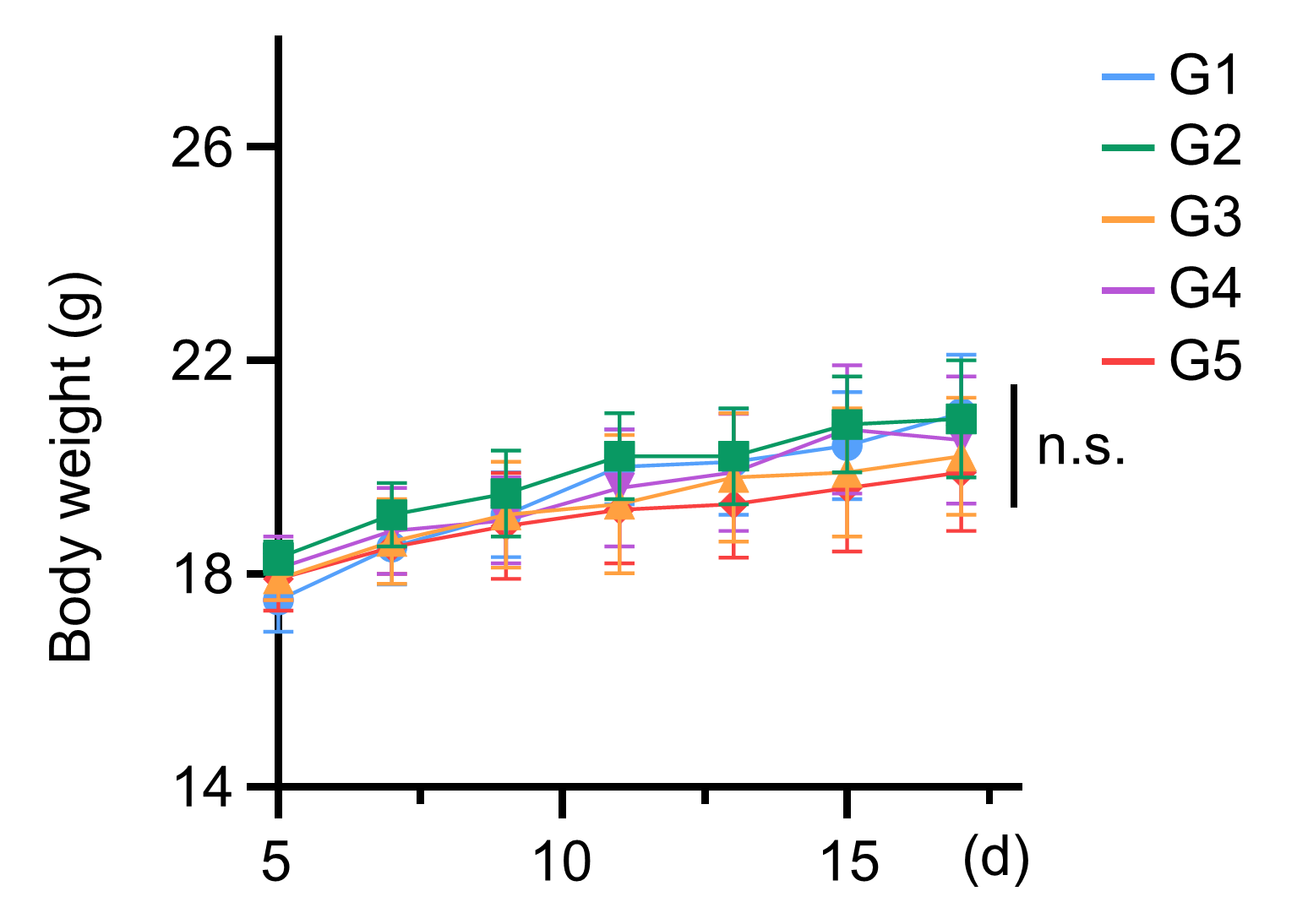


**Fig. S18.** Body weight changes of TC-1-bearing mice after administration of different agents. Data are presented as mean ± SD (*n* = 5). Statistical significance was analyzed by unpaired *t* test. (G1: PBS, G2: MNs(TC-1), G3: MNs(OVs), G4: OVs and G5: MNs(TDEVs@OVs) ).


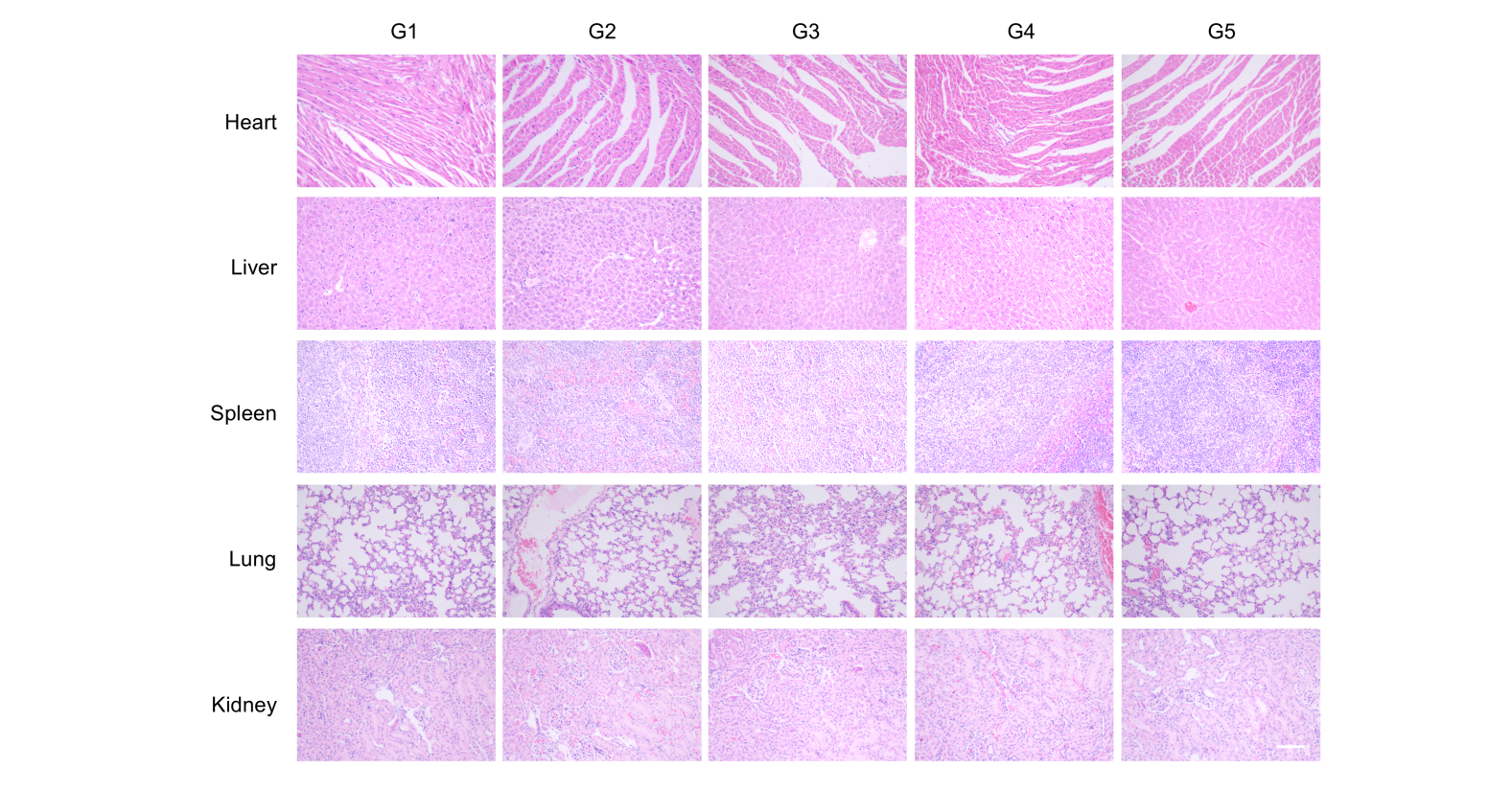


**Fig. S19.** H&E staining images for major organs slices after different treatments. (G1: PBS, G2: MNs(TC-1), G3: MNs(OVs), G4: OVs and G5: MNs(TDEVs@OVs) ). Scale bar: 100 μm.


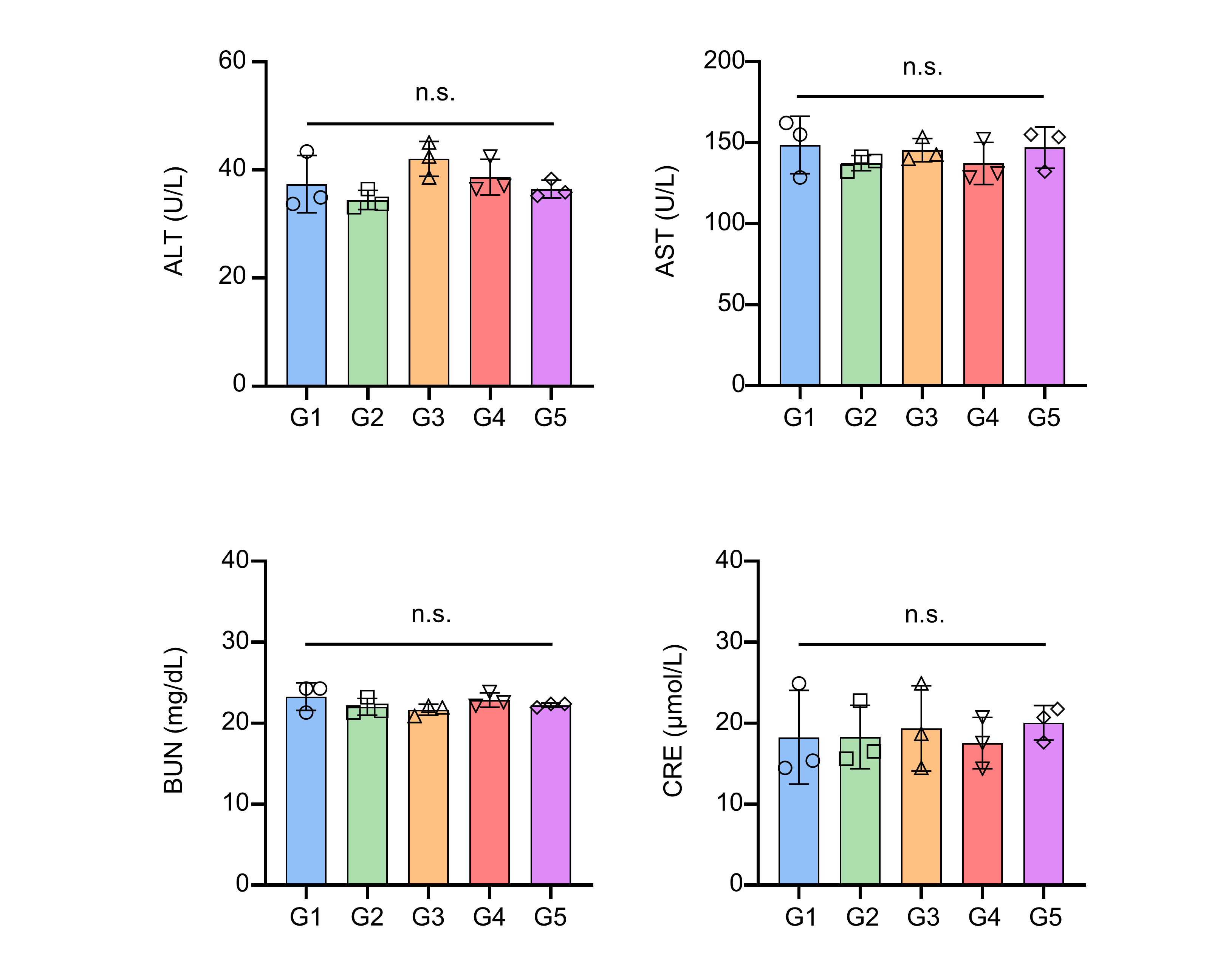


**Fig. S20.** Hepatorenal function indexes including alanine aminotransferase (ALT), aspartate aminotransferase (AST), creatinine (CRE) and blood urea nitrogen (BUN) levels were measured (*n* = 3). Statistical significance was analyzed by unpaired *t* test. (G1: PBS, G2: MNs(TC-1), G3: MNs(OVs), G4: OVs and G5: MNs(TDEVs@OVs) ).


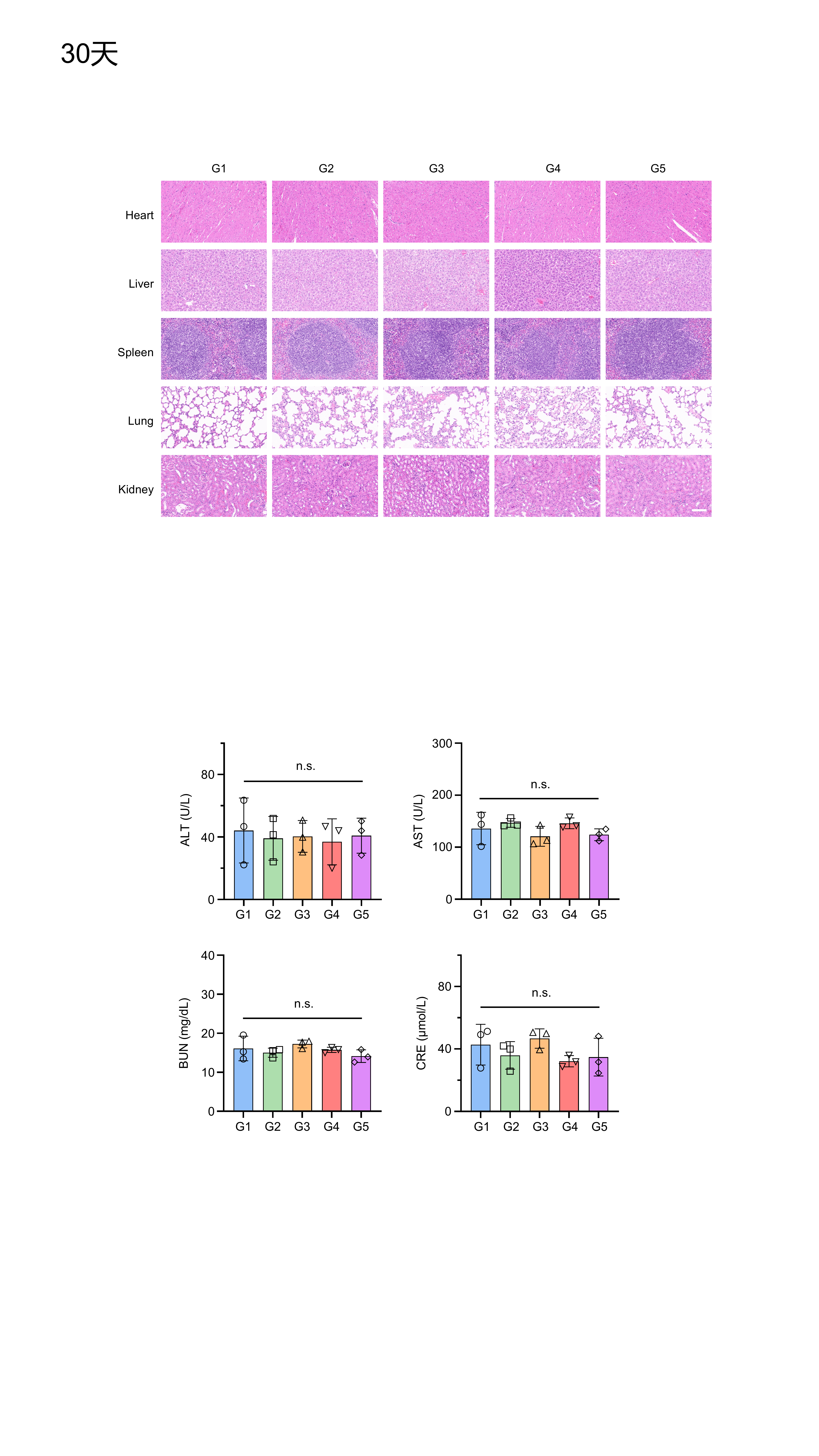


**Fig. S21.** Long-term changes in H&E staining images for major organs slices after different treatments at 30 days after drug administration. (G1: PBS, G2: MNs(TC-1), G3: MNs(OVs), G4: OVs and G5: MNs(TDEVs@OVs) ). Scale bar: 100 μm.


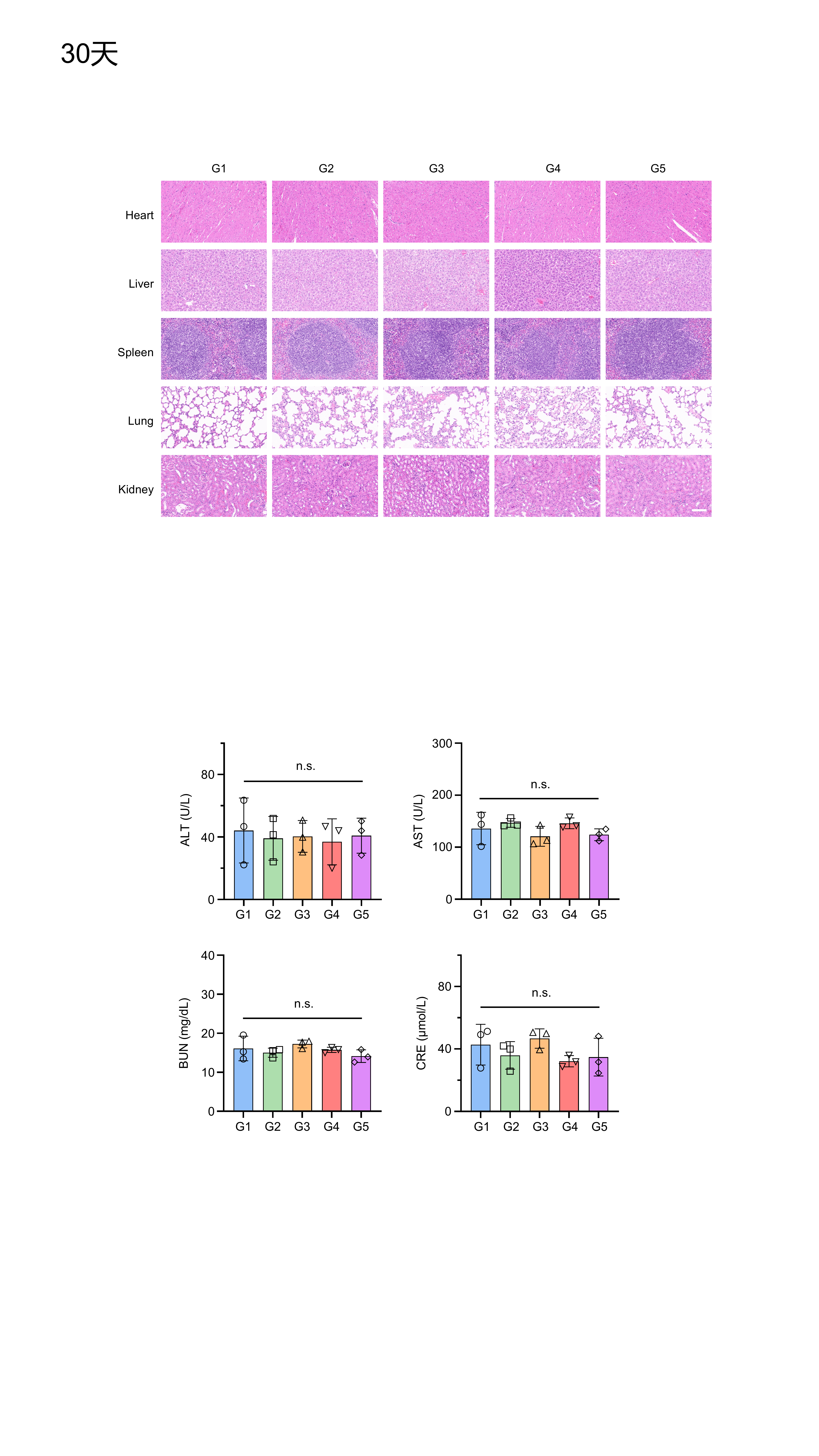


**Fig. S22.** Long-term changes in hepatorenal function indexes including ALT, AST, CRE and BUN levels were measured at 30 days after drug administration (*n* = 3). Statistical significance was analyzed by unpaired *t* test. (G1: PBS, G2: MNs(TC-1), G3: MNs(OVs), G4: OVs and G5: MNs(TDEVs@OVs) ).


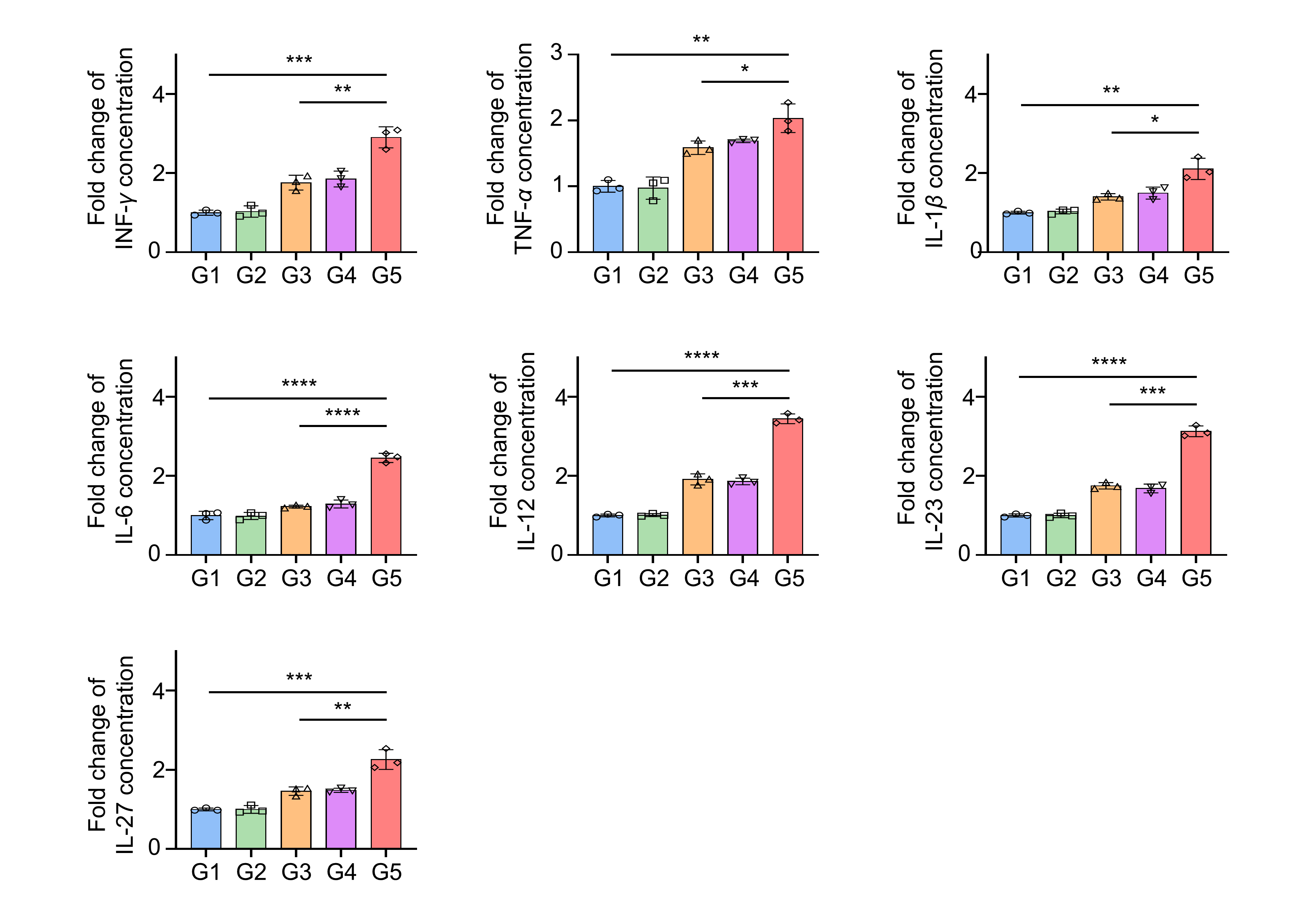


**Fig. S23.** The cytokines in the tumor were detected after different treatments, using the Luminex bead-based ELISA kit (*n* = 3). Statistical significance was analyzed by unpaired *t* test. Data are presented as mean ± SD. *P*-value: **P* < 0.05, ***P* < 0.01, ****P* < 0.001, *****P* < 0.0001. (G1: PBS, G2: MNs(TC-1), G3: MNs(OVs), G4: OVs and G5: MNs(TDEVs@OVs) ).


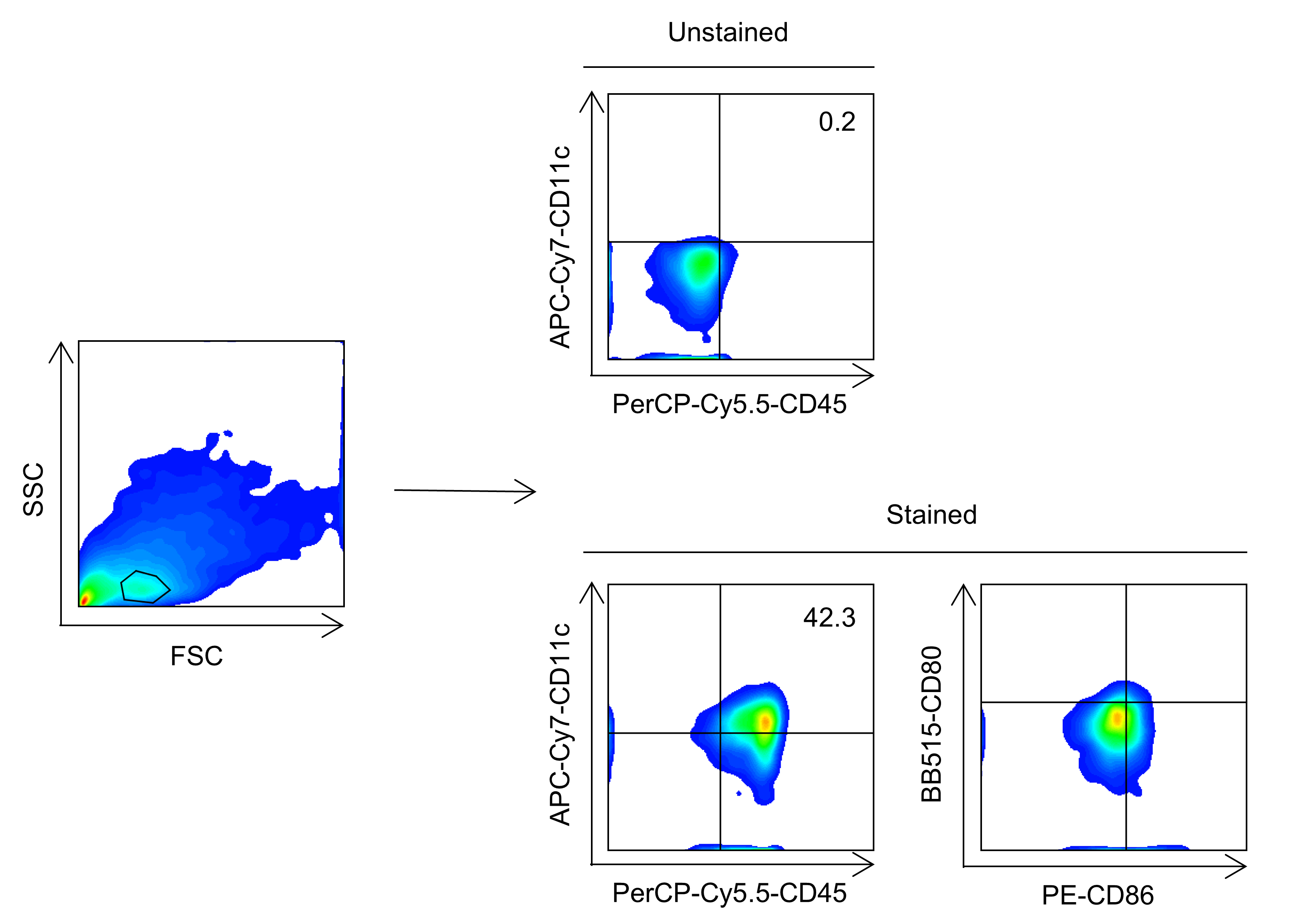


**Fig. S24.** The gating strategy of DCs (CD45^+^CD11c^+^CD80^+^CD86^+^) in the tumor.


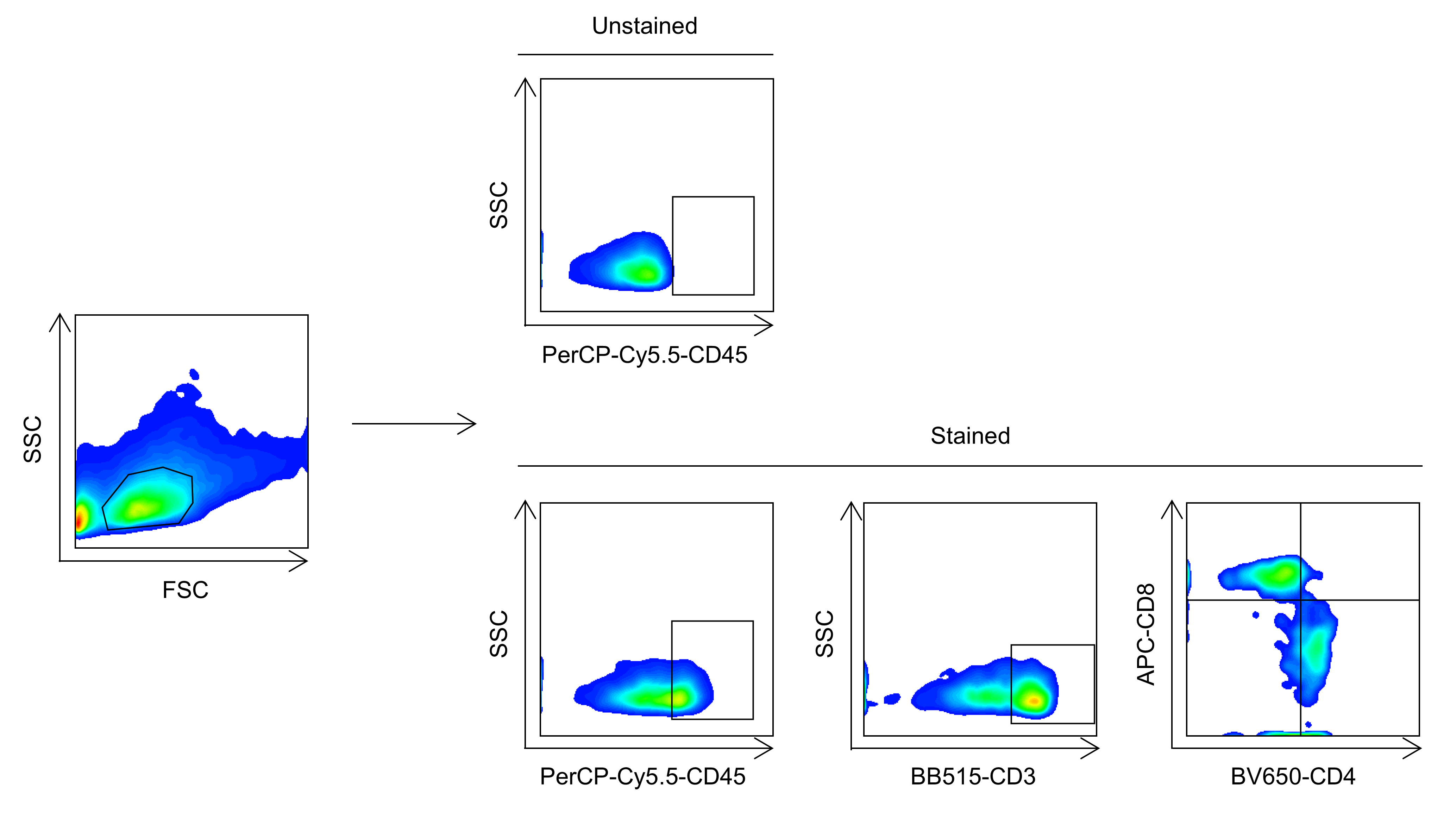


**Fig. S25.** The gating strategy of CD8^+^ T cells (CD45^+^CD3^+^CD8^+^) in the tumor.


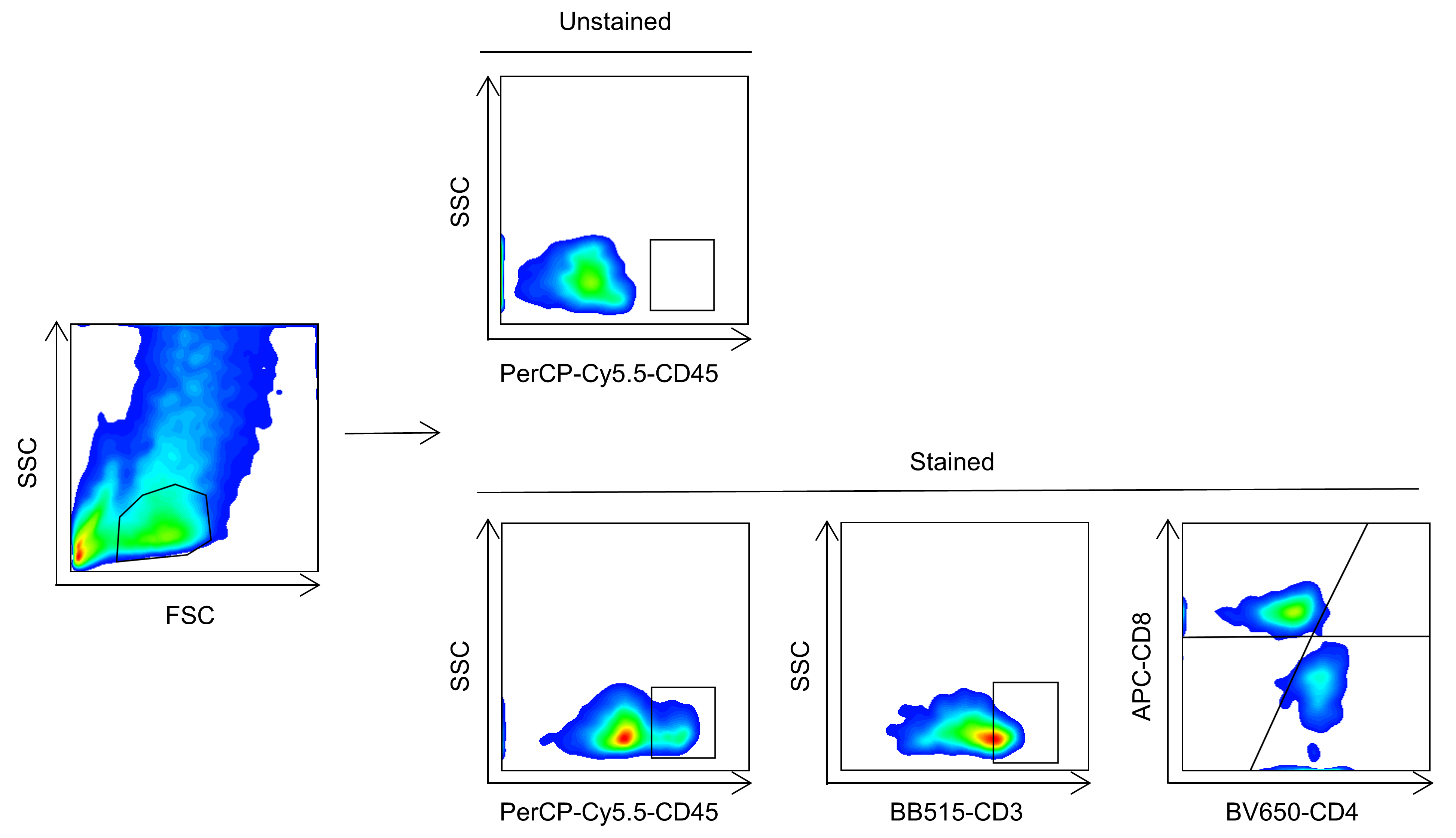


**Fig. S26.** The gating strategy of CD8^+^ T cells (CD45^+^CD3^+^CD8^+^) in the spleen.


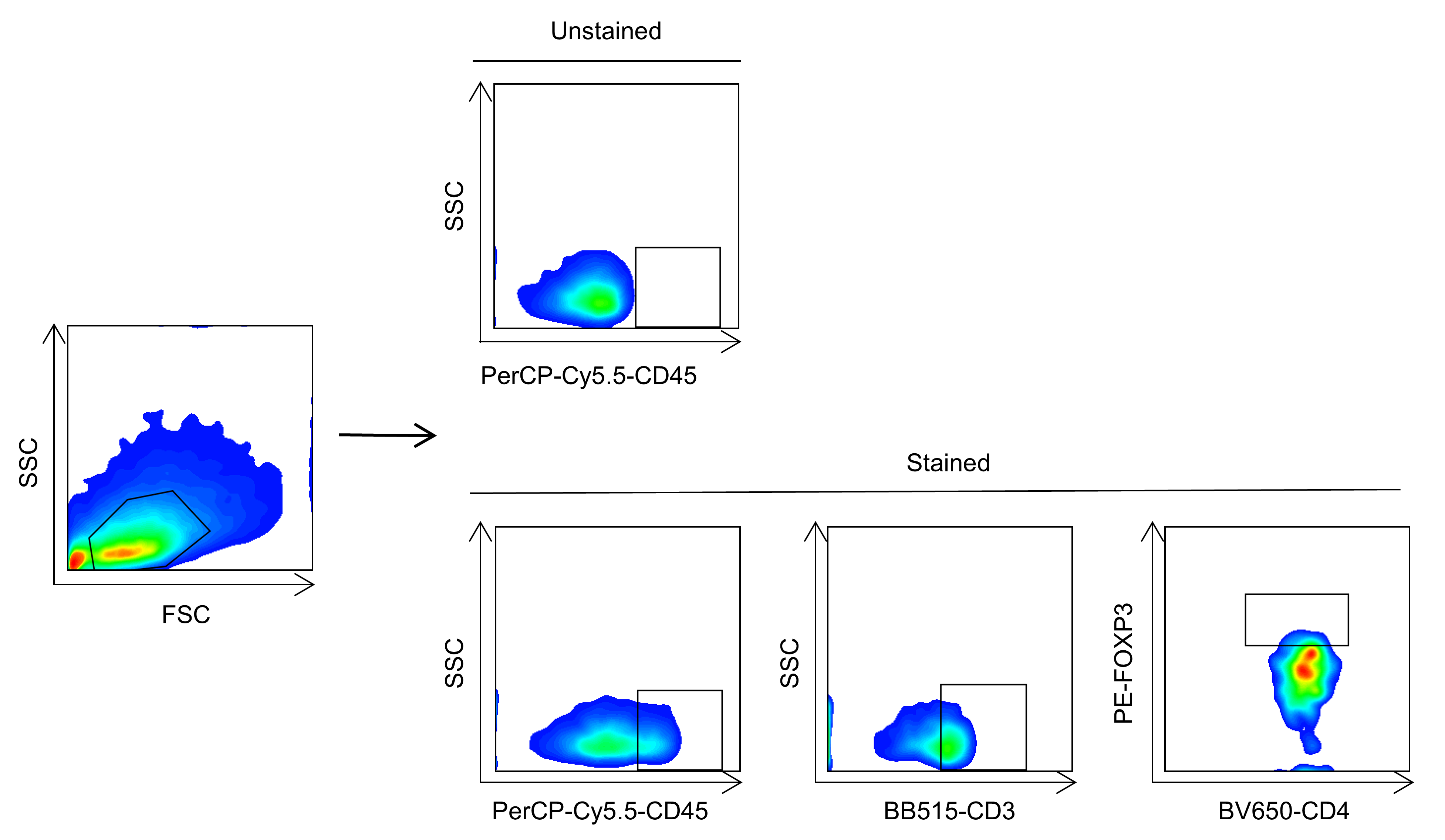


**Fig. S27.** The gating strategy of Treg cells (CD45^+^CD3^+^CD4^+^FOXP3^+^) in the tumor.


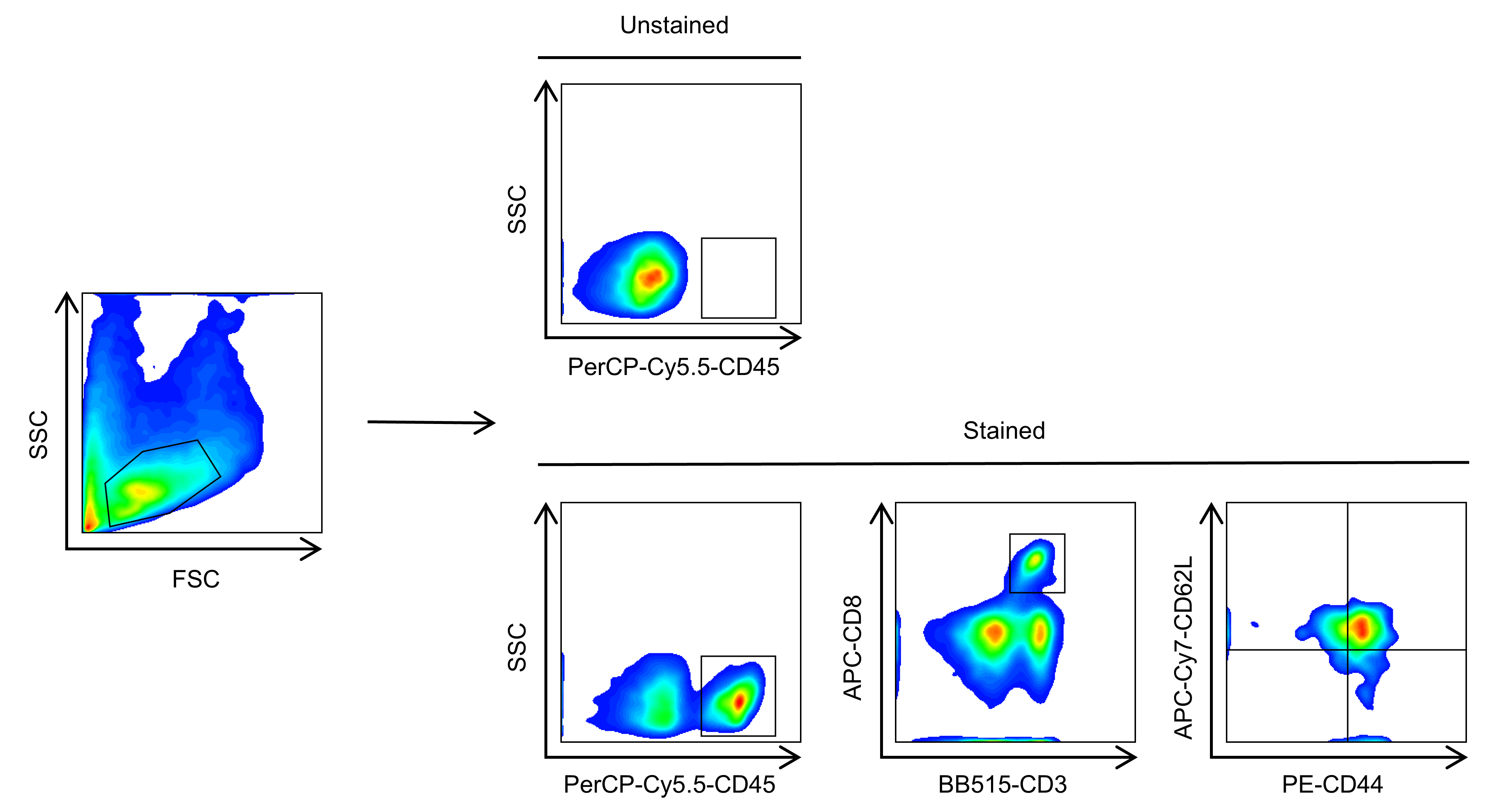


**Fig. S28.** The gating strategy of effector memory T cells (CD45^+^CD3^+^CD8^+^CD62L^-^CD44^+^) in the blood.


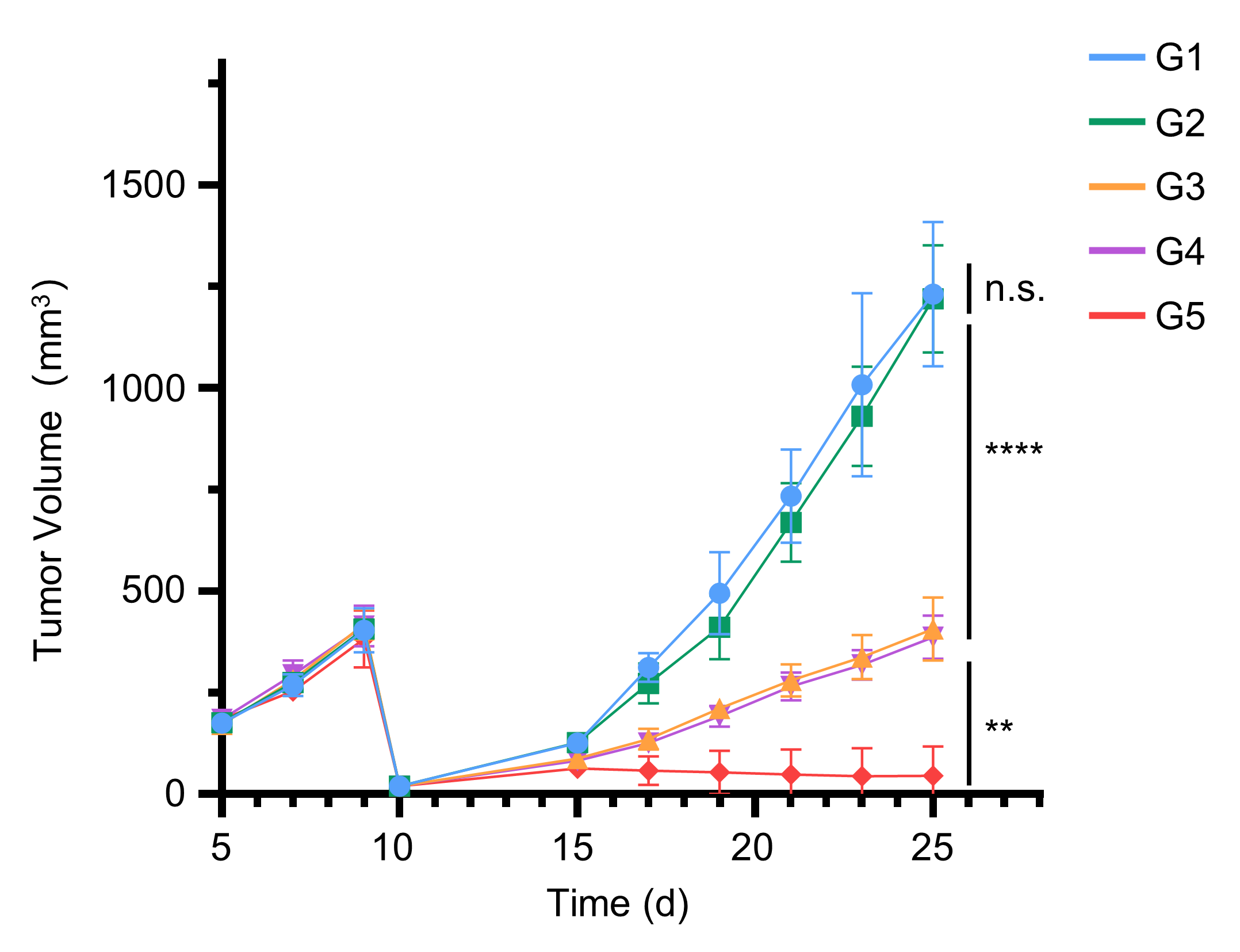


**Fig. S29.** Growth curve of primary tumor volume for TC-1-bearing mice after receiving treatments with different agents (*n* = 5). Data are presented as mean ± SD. Statistical significance was analyzed by unpaired *t* test. *P*-value: ***p* < 0.01, *****p* < 0.0001. (G1: PBS, G2: MNs(TC-1), G3: MNs(OVs), G4: OVs, and G5: MNs(TDEVs@OVs) )


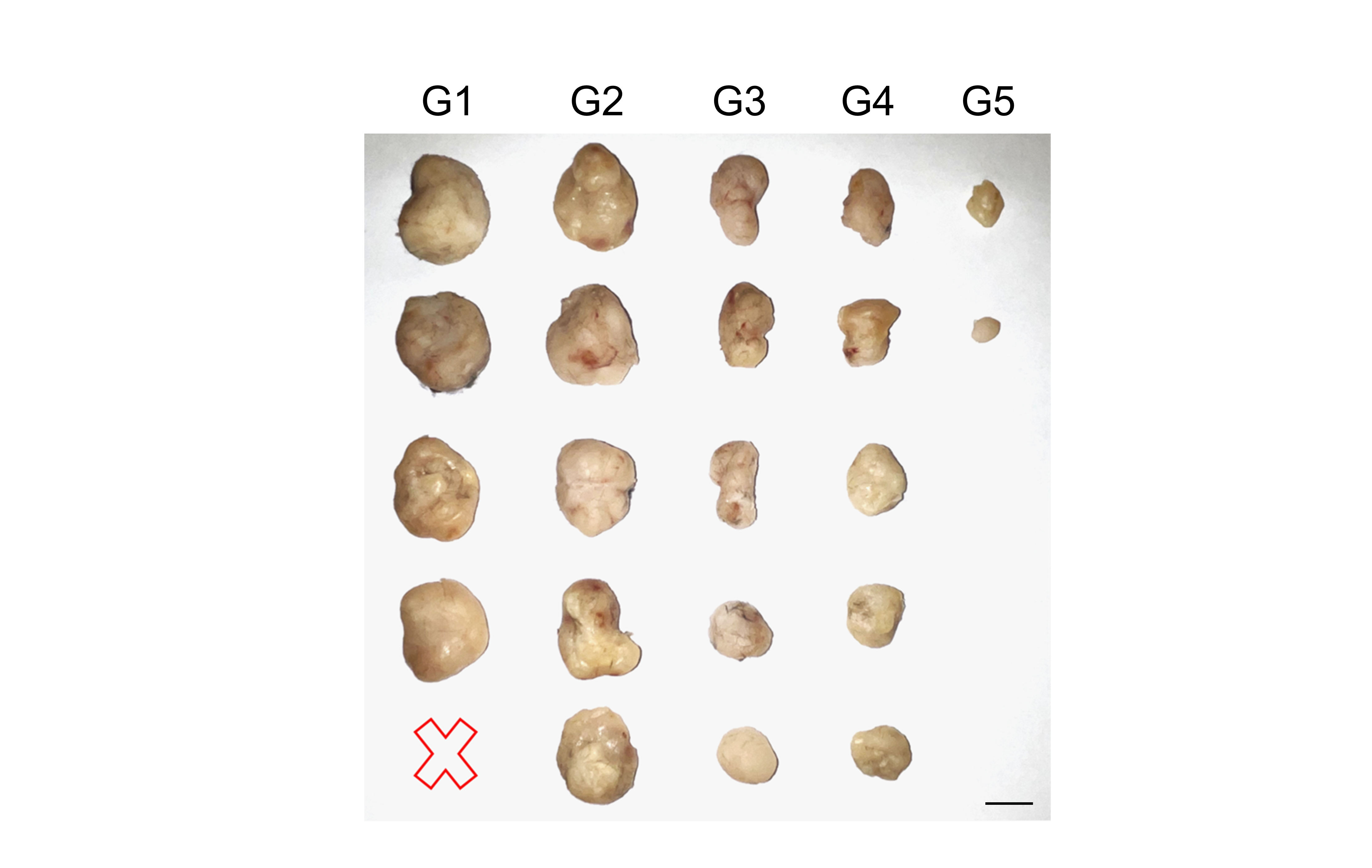


**Fig. S30.** Images of representative primary tumors of different treated groups on the 29th day (*n* = 5). Scale bar: 10 mm. (G1: PBS, G2: MNs(TC-1), G3: MNs(OVs), G4: OVs and G5: MNs(TDEVs@OVs) ).


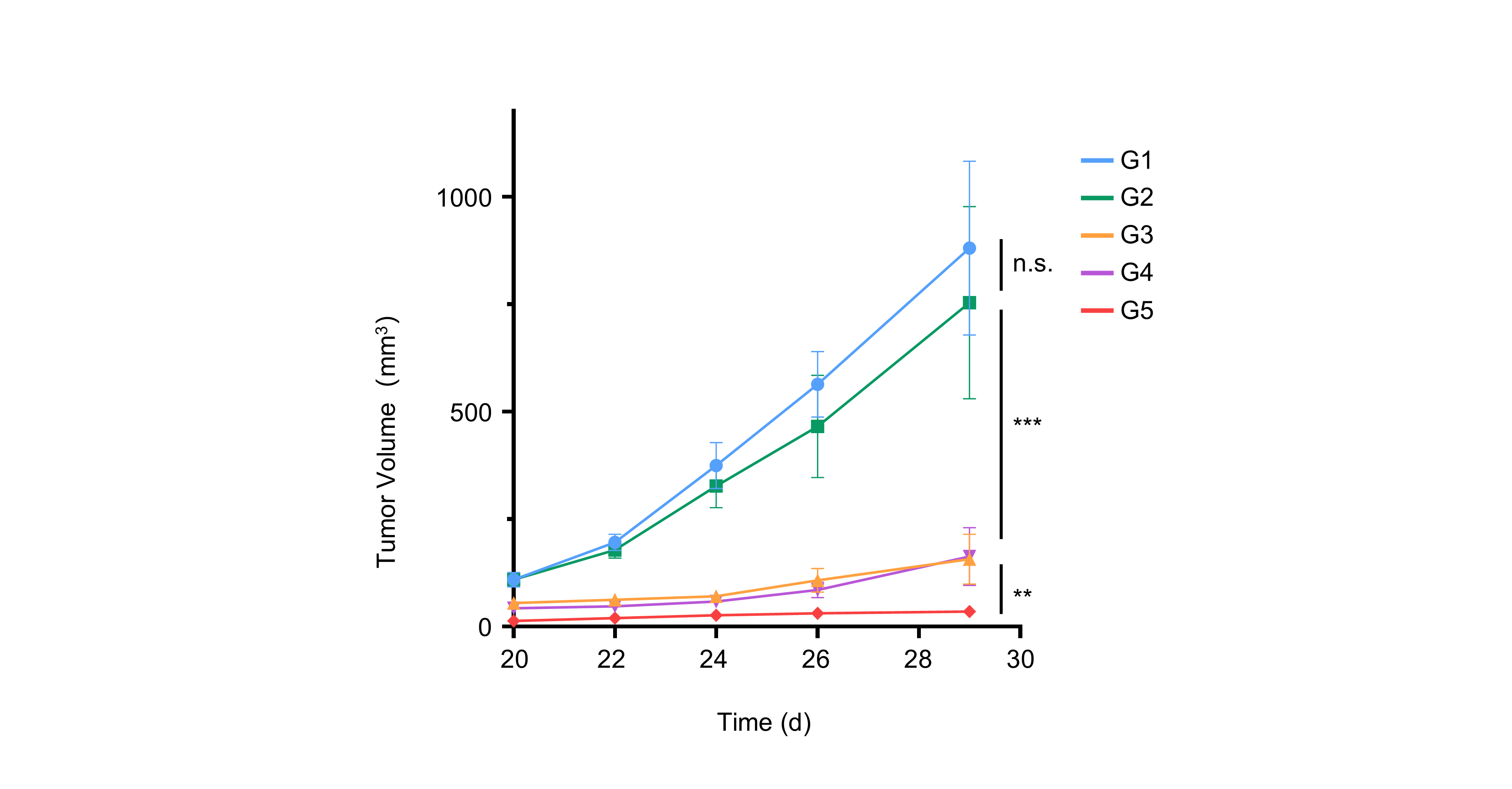


**Fig. S31.** Growth curve of distant tumor volume for TC-1-bearing mice after receiving treatments with different agents (*n* = 5). Data are presented as mean ± SD. Statistical significance was analyzed by unpaired *t* test. *P*-value: ***p* < 0.01, ****p* < 0.001. (G1: PBS, G2: MNs(TC-1), G3: MNs(OVs), G4: OVs and G5: MNs(TDEVs@OVs) ).


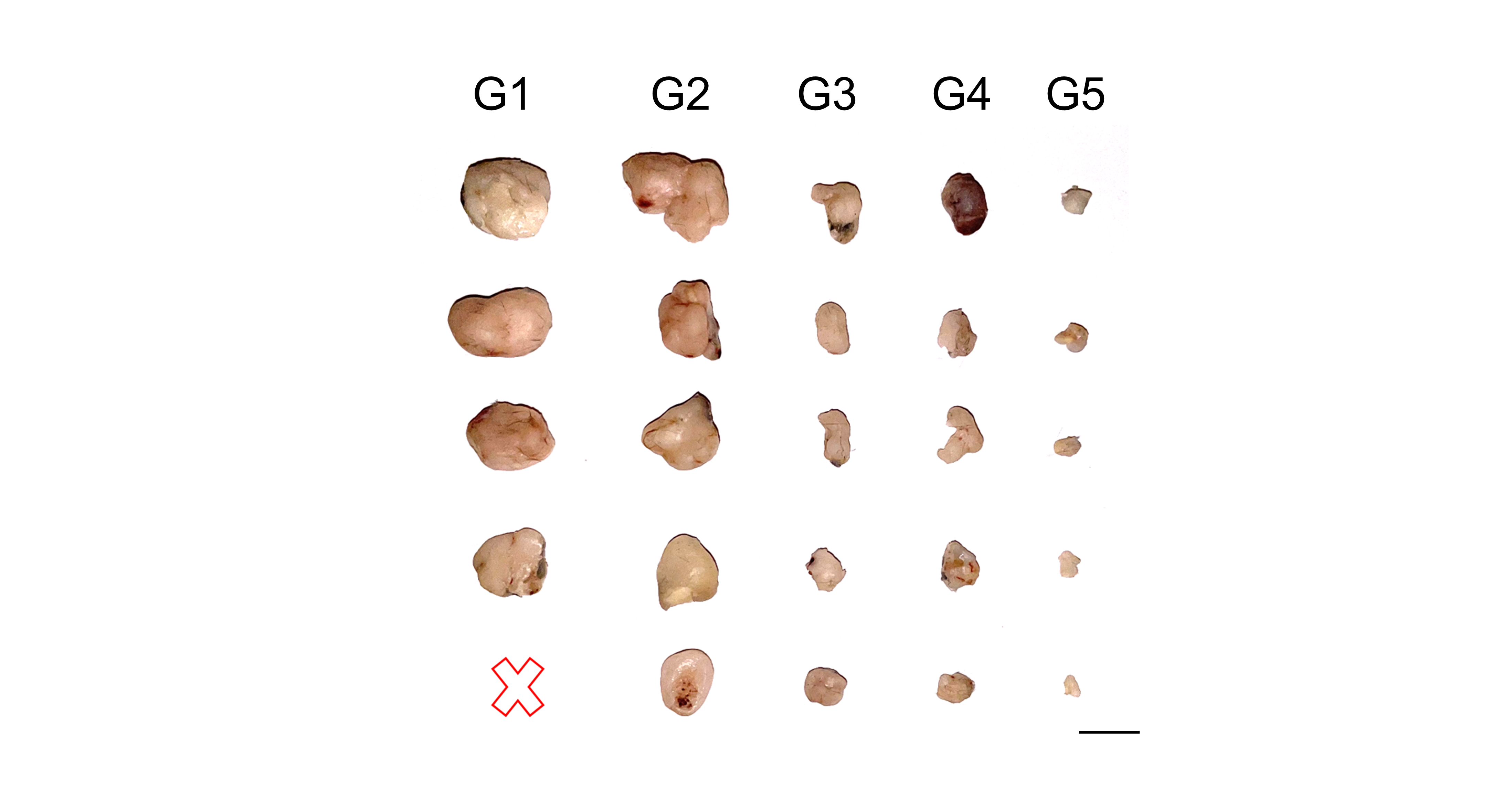


**Fig. S32.** Images of representative distant tumors of different treated groups on the 29th day (*n* = 5); scale bar: 10 mm. (G1: PBS, G2: MNs(TC-1), G3: MNs(OVs), G4: OVs and G5: MNs(TDEVs@OVs) ).


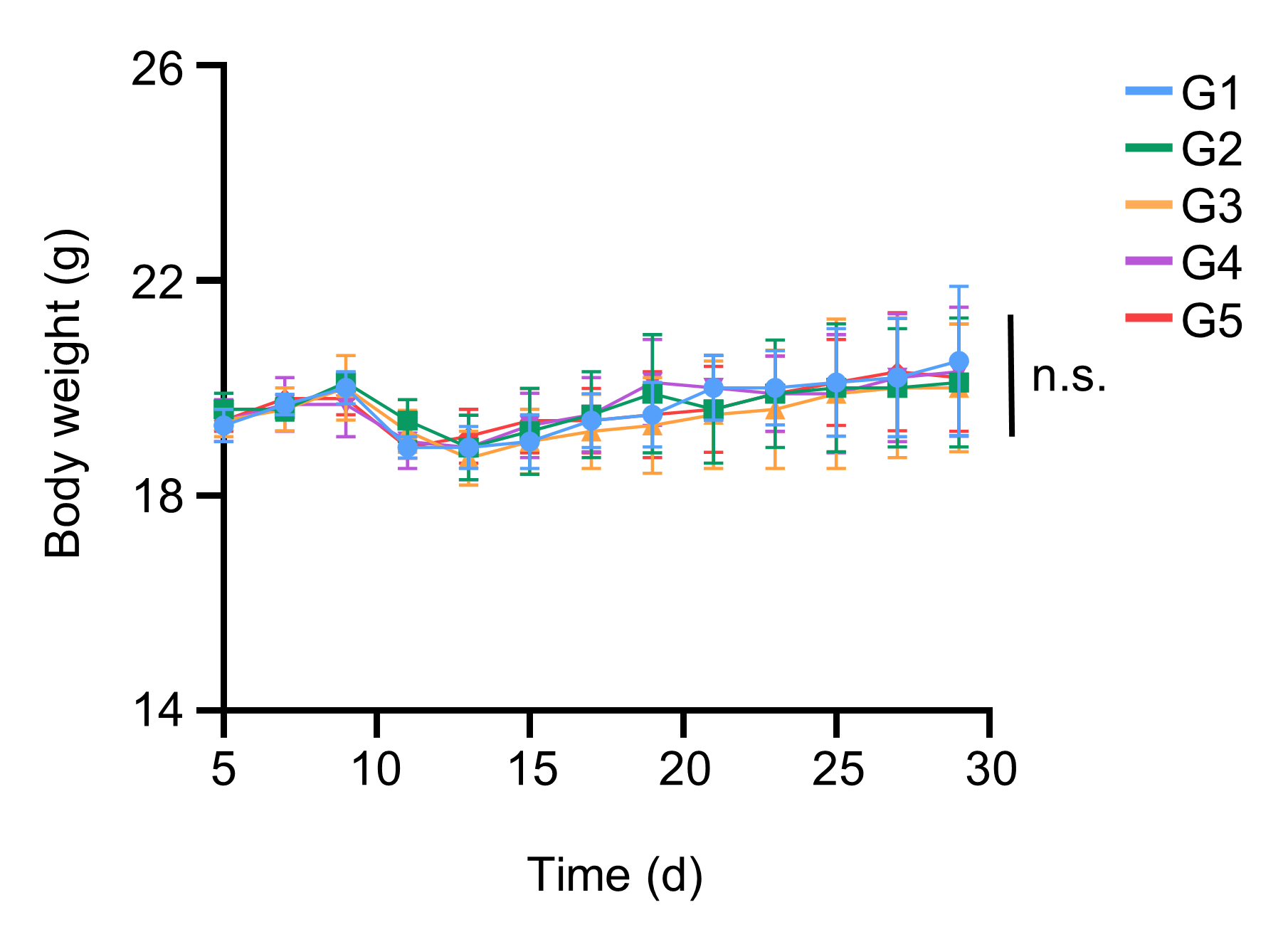


**Fig. S33.** Body weight changes of TC-1-bearing mice after administration of different agents. Data are presented as mean ± SD (*n* = 5). Statistical significance was analyzed by unpaired *t* test. (G1: PBS, G2: MNs(TC-1), G3: MNs(OVs), G4: OVs and G5: MNs(TDEVs@OVs) ).
